# Macro and micro analysis on coal-bearing soil slopes instability based on CFD-DEM coupling method

**DOI:** 10.1371/journal.pone.0257362

**Published:** 2021-09-17

**Authors:** Hong Zhang, Bang Zhang, Can Wu, Kun Chen

**Affiliations:** School of Civil and Architectural Engineering, Nanchang Institute of Technology, Nanchang, China; China University of Mining and Technology, CHINA

## Abstract

By combining the discrete element method (DEM) with computational fluid dynamics (CFD), this study proposes a three-dimensional CFD–DEM fluid–solid coupling microscopic computational model for analyzing the micromechanisms of instability and failure in a coal-bearing soil slope during rainfall. The CFD–DEM fluid–solid coupling model indicated that the main failure mode of the coal-bearing soil slopes was rainwater washing, and the slope sliding surface was predicted as an approximately linear segment. The adaptability of this numerical method was verified by comparing its results with those of rain-washed slopes in an outdoor model test. Rainfall changed the microscopic parameters such as the force chain, coordination number, and porosity of the slope soil particles. The porosity of the slope’s top particles increased from 0.35 in the initial state to 0.80 in the unstable state. This change was directly related to the macroscopic mechanics of the slope soil. By analyzing the changes in the microscopic parameters of the particles, the failure evolution law of the coal-bearing soil slopes during rainfall was explored from a microscopic perspective. This study not only provides a theoretical basis for the protection design and construction of coal-bearing soil slopes in the region but can also analyze macroscopic mechanical laws of discrete media from a micro–macro perspective in geotechnical engineering.

## Introduction

Rainwater infiltrates the soil pores on sloped ground. When the soil pores are completely filled with rainwater, the soil becomes saturated, and its shear strength and stability are reduced. Traditionally, saturated soils are modeled by continuous coupling models, which are usually based on homogenizing mixture theories such as Biot theory or the micromechanical equations of motion [1, 2]. These models require a constitutive relation to describe the stress–strain relation of the solid phases. The most representative constitutive relations are the hat model, the multiple-yield surface plasticity model, and the bounding surface plasticity model. Continuous mechanical models of saturated soil are based on phenomenological descriptions of solid–liquid binary phases, and their equations and constitutive relations make several assumptions. For example, the initial local porosity is usually assumed to equal the overall average porosity, the momentum exchange between phases follows Darcy’s law, and the permeability coefficient is independent of strain. Nevertheless, Darcy’s law is essentially a low-order Navier–Stokes equation, which is functional only for laminar pore flows and negligible inertial forces [3]. High hydraulic gradients can cause nonlaminar motions, and increased volumetric deformation of the soil skeleton can change the porosity, which can also lead to deviations from Darcy’s law [4]. Therefore, a fluid–solid coupling micromechanical model of slopes should be developed from a micro–macro perspective. During rainfall, the model demonstrates the discrete skeleton deformation, pore fluid flow, and mutual multiscale and multiphase couplings.

The Euler–Lagrange method usually treats the rock and soil masses as discrete particles with specific sizes and shapes, and the continuous fluid is divided into several grid units. The average characteristics of the fluid in a grid unit are considered to characterize the entire unit. Averaging avoids the computational inefficiency of calculating the microscopic pore water movements between the soil particles. In such methods, the discrete media and fluids are usually modeled with the discrete element method (DEM) and computational fluid dynamics (CFD), respectively. In the early days, CFD–DEM fluid–solid coupling methods were mainly applied in the industrial field. For example, Tsuji et al. [5] coupled CFD and DEM to simulate the behavior of gas flows in solid particles. In the dynamic collision model of Xu and Yu [6], the CFD and DEM were performed on the same temporal and spatial scales, and the fluid–particle interactions were described by Newton’s third law. Subsequently, Xu et al. [7] and Yu and Xu [8] improved the simulations of movements between solid and fluid phases. Other researchers have also adopted the CFD–DEM coupling method in relevant researches [9–21].

However, the geotechnical engineering community has been slow to adopt the CFD–DEM fluid–solid coupled model. Shamy and Zeghal [22] applied this method to geotechnical analyses. They simplified the Navier–Stokes equation and analyzed the seepage through slope soil using a three-dimensional (3D) CFD–DEM coupling method. In simulations, their method effectively captured the micromechanism of seepage through the soil. Similar applications of the CFD–DEM coupling method have obtained favorable results in other soil seepage and liquefaction studies [23–27]. Recently, Jiang and Zhang [28] and Khalili and Mahboubi [29] embedded discrete CFD–DEM control equations into the commercial software PFC^2D^. They numerically simulated single-particle free settling in the water, a one-sided drainage one-bit consolidation test, and a biaxial undrained compression test, reporting ideal results. Wang et al. [30] simulated the effects of soil particle shape using a particle rolling resistance model, which they introduced to the CFD–DEM coupling control equation. They showed that the rolling resistance model well documented the effects of particle shape on the angle of repose of sand piles and the porosity of soil deposits. The above studies suggest that CFD–DEM coupling models are suitable for micro–macro analyses of mutual soil–fluid coupling.

In this study, the research target was a coal-bearing soil slope from the Wanzai–Yichun Expressway Project in Jiangxi Province, China. On the basis of CFD–DEM coupling methods, a 3D CFD–DEM fluid–solid microaction calculation model was established for the soil slope. The failure mechanism of the coal-bearing soil slope during rainfall was analyzed, and the calculation results well agreed with the model test results. The results are expected to provide a theoretical basis for the protection design and construction of coal-bearing soil slopes in this region.

## Research methods

### Basic principle of CFD–DEM fluid–solid coupling calculation

#### Fluid–particle interaction

Fluid–solid coupling theory (CFD–DEM) investigates the effect of fluid on soil particles [31]. In this study, the fluid was regarded as a continuum and the soil was regarded as discontinuous particles. CFD treats the fluid velocity as a macroscopic quantity, specifically, the volume flow rate per unit cross-sectional area. The fluid is assumed to be generated over the whole cross-section, whereas the actual fluid velocity appears only in the particle pore space. Here, the particle–fluid interaction is simplified to the drag force of the fluid on the particles and the fluid–pressure gradient force. This simplification meets our research requirements. The interaction between fluid and particles can be expressed as follows [32]:

$$\frac{\partial \vec{u}}{\partial t}=\frac{{\vec{f}}_{mech}+{\vec{f}}_{fluid}}{m}+\vec{g},$$ (1)

$$\frac{\partial \vec{\omega }}{\partial t}=\frac{M}{I},$$ (2)

where $\vec{u}$ is the particle velocity, *m* is the particle mass, ${\vec{f}}_{fluid}$ is the fluid force (including drag force and fluid–pressure gradient force), ${\vec{f}}_{mech}$ is the sum of the external forces acting on the particles, $\vec{g}$ is the gravitational acceleration, $\vec{\omega }$ is the rotational angular velocity of the particles, *I* is the moment of inertia, and *M* is the force moment acting on the particles.

Assuming that the force exerted by the fluid on the particles always acts on the centroid (ignoring the bending moment), the drag force is given by

$${\vec{f}}_{drag}={\vec{f}}_{0}\varepsilon^{-\chi }\;{\vec{f}}_{drag}={\vec{f}}_{0}\varepsilon^{-\chi },$$ (3)

where ${\vec{f}}_{0}$ is the drag force on a single particle, *ε* is the porosity of the unit in which the fluid is located, and *χ* is an empirical coefficient that renders the drag force applied to both high and low local porosity.

The drag force of a single particle in Eq (3) is given by

$${\vec{f}}_{0}=\frac{1}{2}C_{d}\rho_{f}\pi r^{2}\left|\vec{u}-\vec{v}\right|(\vec{u}-\vec{v}),$$ (4)

where *ρ*_*f*_ is the fluid density, *r* is the particle radius, $\vec{v}$ and $\vec{u}$ are the fluid and particle velocities, respectively, and *μ*_*f*_ is the dynamic viscosity coefficient of the fluid. *C*_*d*_ is the drag force coefficient, which is expressed as follows:

$$C_{d}={\left(0.63+\frac{4.8}{\sqrt{R_{ep}}}\right)}^{2}.$$ (5)

The exponent *χ* in Eq (3) is calculated as

$$\chi =3.7-0.65\exp(-\frac{{\left(1.5-\text{lg}R_{ep}\right)}^{2}}{2}),$$ (6)

where *R*_*ep*_ is the Reynolds number of particles, defined as

$$R_{ep}=\frac{2\rho_{f}r\left|\vec{u}-\vec{v}\right|}{\mu_{f}}.$$ (7)

The force exerted on a unit volume of the fluid is calculated as

$${\vec{f}}_{b}=\frac{\sum_{j}f_{drag}^{j}}{V}=\frac{4}{3}\pi r^{3}(\nabla p-\rho_{f}\vec{g}),$$ (8)

where *V* is the volume of a fluid unit, *∇p* is the fluid gradient, and the sum object on the molecule is the particles superposed by the fluid unit.

The interaction force of the fluid on the particles is then expressed as follows:

$${\vec{f}}_{fluid}={\vec{f}}_{drag}+{\vec{f}}_{b}=\frac{1}{2}\varepsilon^{-\chi }C_{d}\rho_{f}\pi r^{2}|\vec{u}-\vec{v}|(\vec{u}-\vec{v})+\frac{4}{3}\pi r^{3}(\nabla p-\rho_{f}\vec{g}).$$ (9)

#### Fluid–fluid interaction

*Continuum equation*. The average Navier–Stokes equation is useful for meso analyses and quantitative descriptions of pore fluid characteristics. The flow control equation derived from the law of conservation of mass (called the continuum equation) defines the net mass flux through the volume element per unit time. As this quantity equals the change of mass in the volume element, we can write

$$\frac{\partial (\varepsilon \rho_{f})}{\partial t}+\nabla \cdot (\varepsilon \rho_{f}\vec{v})=0$$ (10)

where ∇ is the Laplace operator.

*Momentum equation*. The momentum equation of fluids is a variant of Newton’s second law of motion. It describes the increment of momentum per unit volume per unit time and is contributed mainly by the momentum carried by the surface inflow mass and the momenta caused by various forces (surface stress, fluid pressure, particle drag on the fluid, and volume forces). Averaging the external forces in the volume element, Anderson and Jackson [33] deduced the following momentum equation for fluids:

$$\frac{\partial (\varepsilon \rho_{f})}{\partial t}+\nabla \cdot (\varepsilon \rho_{f}\vec{v}\vec{v})-\varepsilon \nabla \cdot (\mu_{f}\nabla \vec{v})=-\nabla p-{\vec{f}}_{drag}+\varepsilon \rho_{f}g,$$ (11)

where *p* is the fluid pressure and the minus sign denotes a positive force acting on the particles.

#### Particle–motion control equation

The particle motions satisfy Newton’s second law. The fluid–particle interaction force ${\vec{f}}_{fluid}$ is a function of the contact and volume forces. For the *i*th particle, the interaction force is given by

$$m_{i}\frac{\text{d}{\vec{u}}_{i}}{\text{d}t}=\sum_{j=1}^{n_{i}}{\vec{f}}_{ij}+{\vec{f}}_{fluid(i)}+{\vec{f}}_{g(i)}.$$ (12)

The moment of inertia of the *i*th particle is given by

$$I_{i}\frac{\text{d}{\vec{\omega }}_{i}}{\text{d}t}=\sum_{j=1}^{n_{i}}M_{ij}.$$ (13)

In these expressions, ${\vec{u}}_{i}$, ${\vec{f}}_{ij}$, and ${\vec{f}}_{g(i)}$ denote the velocity, acting contact force, and gravity of the *i*th particle, respectively, and *M*_*ij*_ is the moment of inertia of the *i*th particle when impacted by the *j*th particle.

#### CFD–DEM coupling process and program implementation

**Fig 1:**
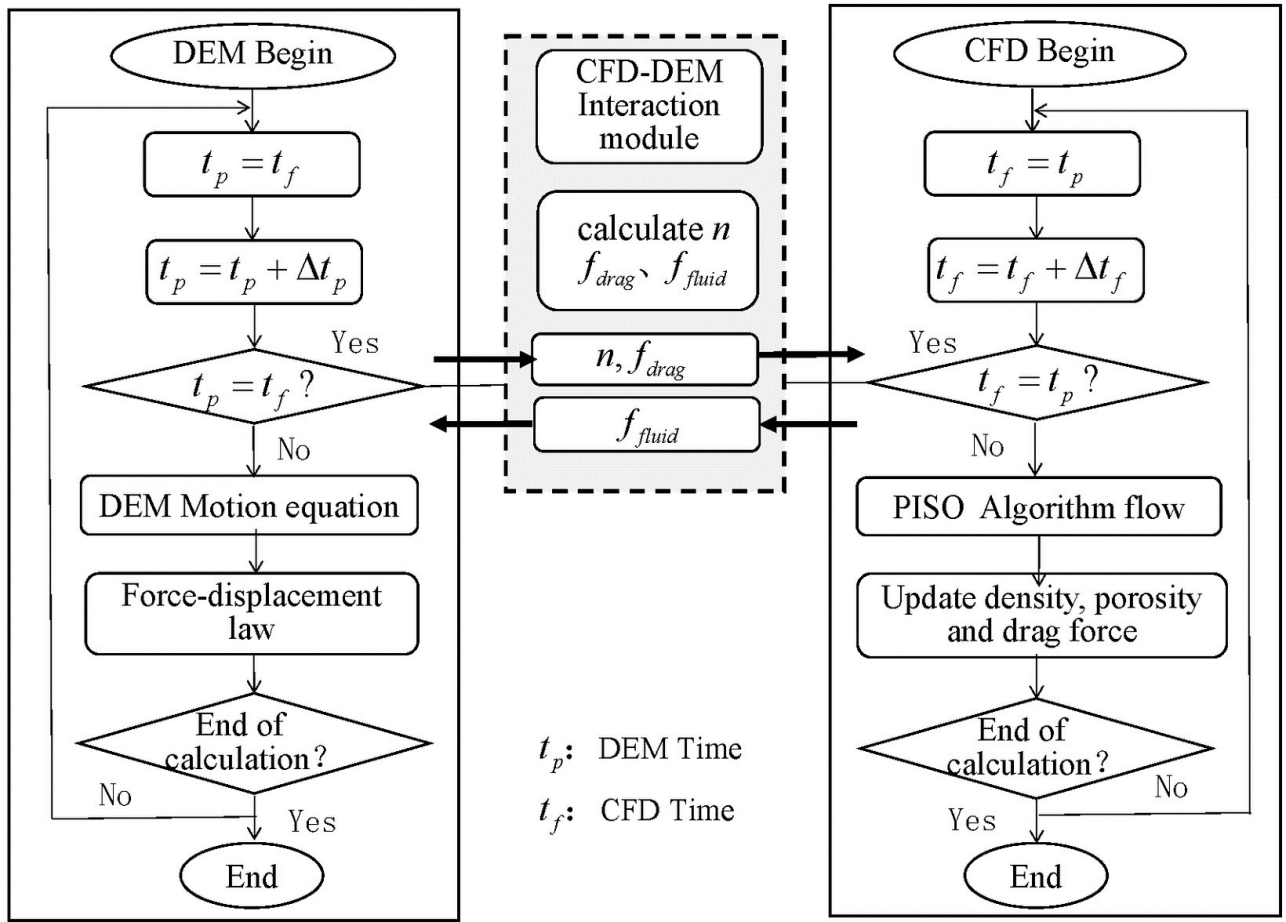
CFD-DEM coupling calculation flow.

The program was composed in the fish language of the business software PFC^3D^, which enables data exchange between PFC^3D^ and its embedded CFD solver and hence the coupling calculations of fluid and solid. The fluid control Eqs (10) and (11) in the CFD module are first discretized using the finite volume method under the specified boundary conditions, and the discrete equations are solved by the pressure splitting of operators stress–velocity coupling algorithm [34]. Meanwhile, the grid data are sent to the DEM module, which calculates the porosity and drag force and returns the data to the CFD module. Next, the force between the fluid and the particles in each time step control unit is sent to the DEM calculation module, and the fluid force is then applied to the soil particles using the DEM. At this time, the mechanics between the particles are also calculated. Finally, the fluid forces and porosity are returned to the CFD model, and the calculations are iterated until the end of the program. Fig 1 presents a flow chart of the CFD–DEM coupling calculation. When the computational time of CFD is equal to that of DEM, the data interaction step is triggered to complete the fluid–particle interaction calculation.

*Verifying the fluid–solid coupling program of CFD–DEM*. According to Stokes’ law, a small ball immersed in water will eventually settle under the action of gravity. This fundamental principle is exploited in the densitometer method, which measures the gradation of fine-grained soil in geotechnical tests. In this subsection, the feasibility of the above fluid–solid coupling program is tested on the free-falling motion of a single particle under the action of gravity in water.

The particles fell freely in the liquid, and their velocity became stable after a certain time. The solid particles in the fluid were affected by gravity, buoyancy, and drag forces. The motion equation of falling spherical particles is expressed as follows:

$$\frac{4}{3}\pi r^{3}\rho_{p}\frac{\text{d}{\vec{u}}_{z}}{\text{d}t}=\frac{4}{3}\pi r^{3}(\rho_{p}-\rho_{f})\vec{g}-\frac{1}{2}\pi r^{2}\rho_{f}C_{d}{\vec{\mu }}_{z}^{2},$$ (14)

where *ρ*_*p*_ and *ρ*_*f*_ are the particle and fluid densities, respectively; *r* is the particle radius; ${\vec{\mu }}_{z}$ is the vertical velocity of the falling particle; and *C*_*d*_ is the drag coefficient.

At the (constant) settling velocity of the particles, $\frac{\text{d}{\vec{u}}_{z}}{\text{d}t}$ is 0, and Eq (14) simplifies to

$$\frac{4}{3}\pi r^{3}(\rho_{p}-\rho_{f})\vec{g}=\frac{1}{2}\pi r^{2}\rho_{f}C_{d}{\vec{\mu }}_{z}^{2}.$$ (15)

When the Reynolds number *R*_*ep*_ is very small, the drag coefficient *C*_*d*_ simplifies to

$$C_{d}=\frac{24}{R_{ep}}.$$ (16)

Therefore, the vertical velocity of the falling particle ${\vec{u}}_{z}$ can be solved by Stokes law and is given as

$${\vec{u}}_{z}=\frac{2}{9}\frac{r^{2}(\rho_{p}-\rho_{f})\vec{g}}{\mu_{f}}.$$ (17)

In this test, two particles of different radii (1.5 and 2.0 mm) freely sank under the action of gravity. The static viscosity of the fluid was 1.0 **Pa · s**, and the particle and fluid densities were 2400 and 1000 kg/m^3^, respectively. Substituting the above parameters into Eq (17), the final settling velocities of the 1.5- and 2.0-mm-radius particles were calculated as −6.86 and −12.1 mm/s, respectively.

**Fig 2:**
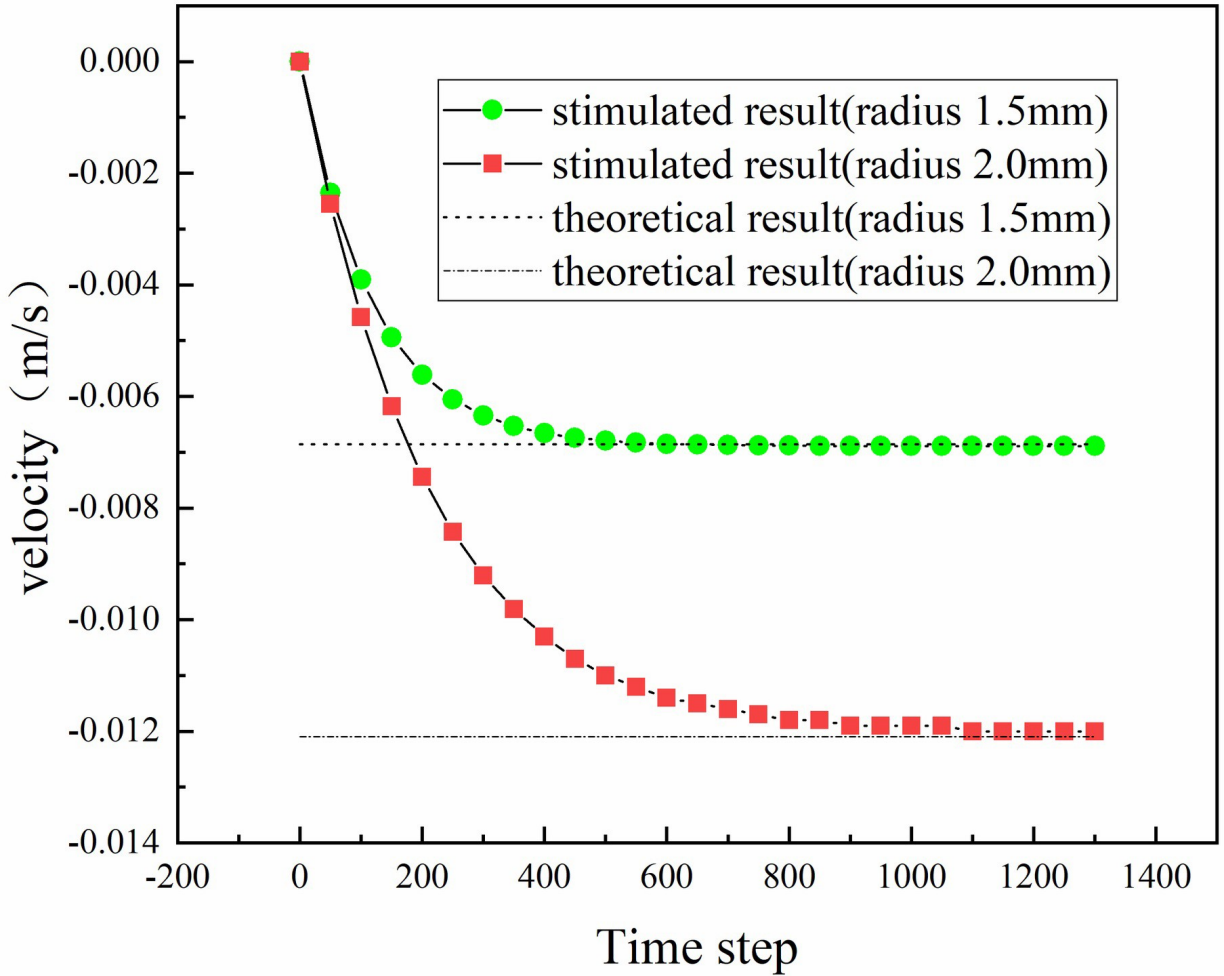
Velocity of falling particles.

After opening the CFD–DEM coupling program module on the PFC software platform, the grid data were imported and the above fluid parameters were specified. Particles of the same size and mass were generated in the fluid at the same time. As they sank under the action of gravity, the 1.5- and 2.0-mm-radius particles tended to final velocities of −6.89 and −12 mm/s, respectively, close to the theoretical results (see Fig 2). Substituting the calculated particle velocities into Eq (7), the Rayleigh numbers were calculated as 0.002067 and 0.0048, respectively, far less than 1, thus meeting the requirements of the theoretical calculation. This result confirms that fluid–particle interactions can be realized by the developed fluid–solid coupling program.

### Coupled CFD–DEM numerical simulations of coal-bearing soil slope instability

#### Outdoor test model of coal-bearing soil slope

**Fig 3:**
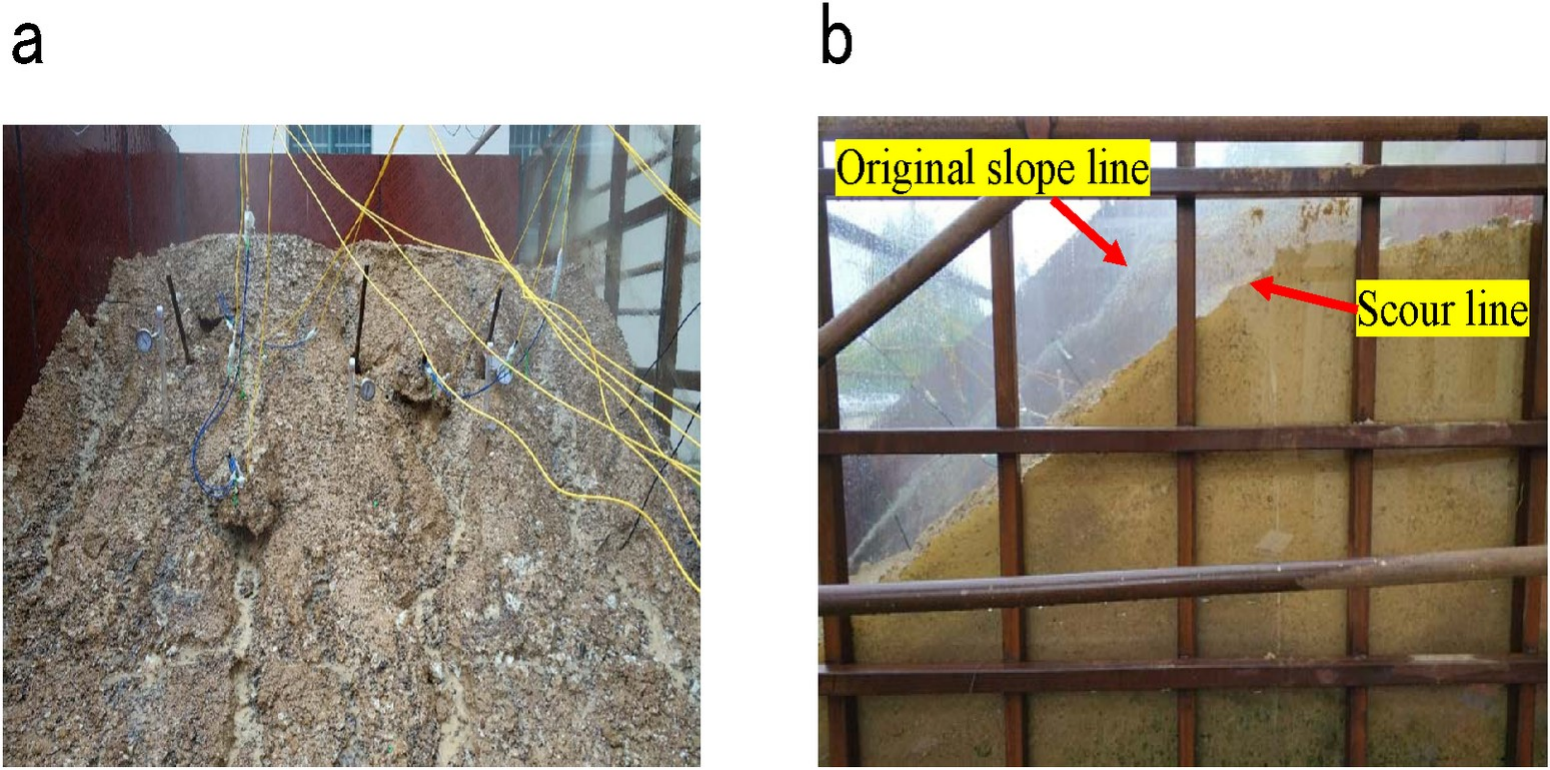
The site of slope failure test. (a) Front of slope model. (b) Side of slope model.

To identify the failure mechanism of coal-bearing soil slopes during rainfall infiltration, the project team conducted outdoor slope model tests under artificial rainfall. A slope model was constructed in a box of dimensions (4.5 × 3.0 × 2.4) m^3^ (length × width × height). The test soil samples were taken from remolded soil exposed during excavations on a coal-bearing soil slope at K30 + 120 in section A5 of the Wanzai–Yichun Expressway Project in Jiangxi Province, China. The slope model was filled with a soil layer at 40 cm intervals at a slope ratio of 1:1.5. The simulated rainfall intensity was set to 9.84 × 10^−7^ m/s, the average intensity of the maximum rainfall in Yichun, Jiangxi Province, during July and August of the most recent decade. The tests were conducted on November 19, 2018, from 8:00 am to 6:00 pm. Field observations (Fig 3) showed that water and soil loss, along with rainwater erosion, were the main failure modes of slope stability.

**Fig 4:**
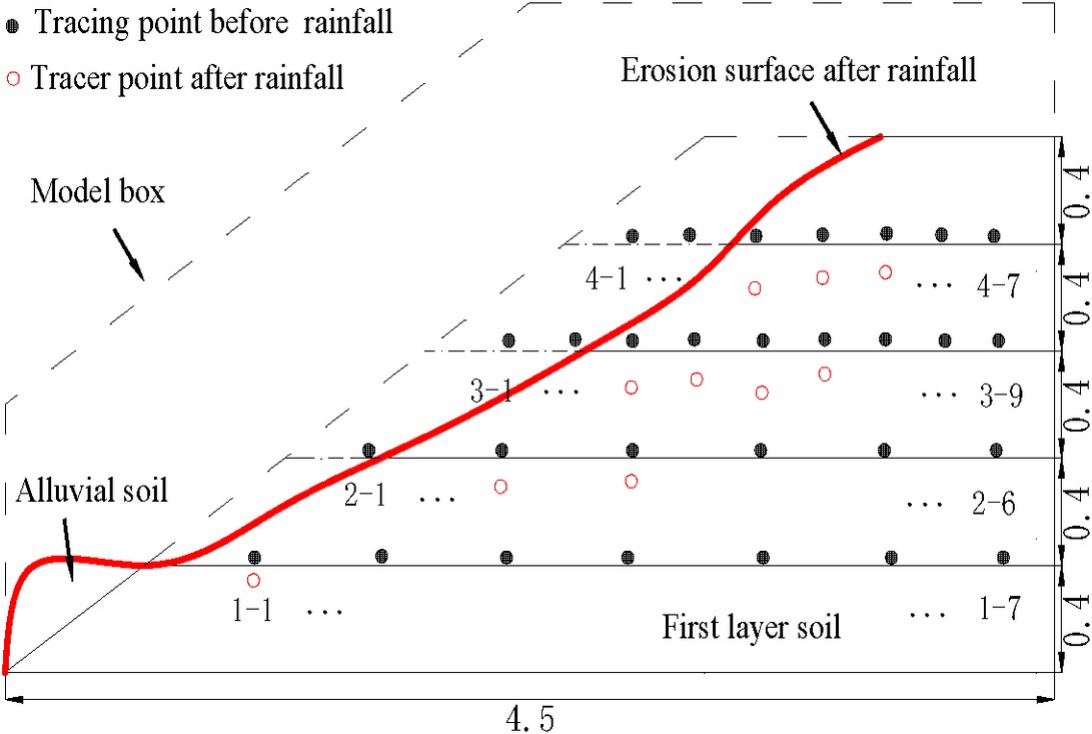
Schematic diagram of soil movement of tracer point on the site of slope failure test (unit: M).

*Soil displacement monitoring of model slope*. During the test, 29 side tracer points in four rows were buried in the slope soil (Fig 4), and their movements were clearly visible through the Plexiglas side of the model box. During continuous rainfall, the main failure mode of the soil (rain-wash) formed erosion ditches at different depths from the slope surface. The maximum depth of these ditches was 0.36 m in the mid-upper slope.

**Fig 5:**
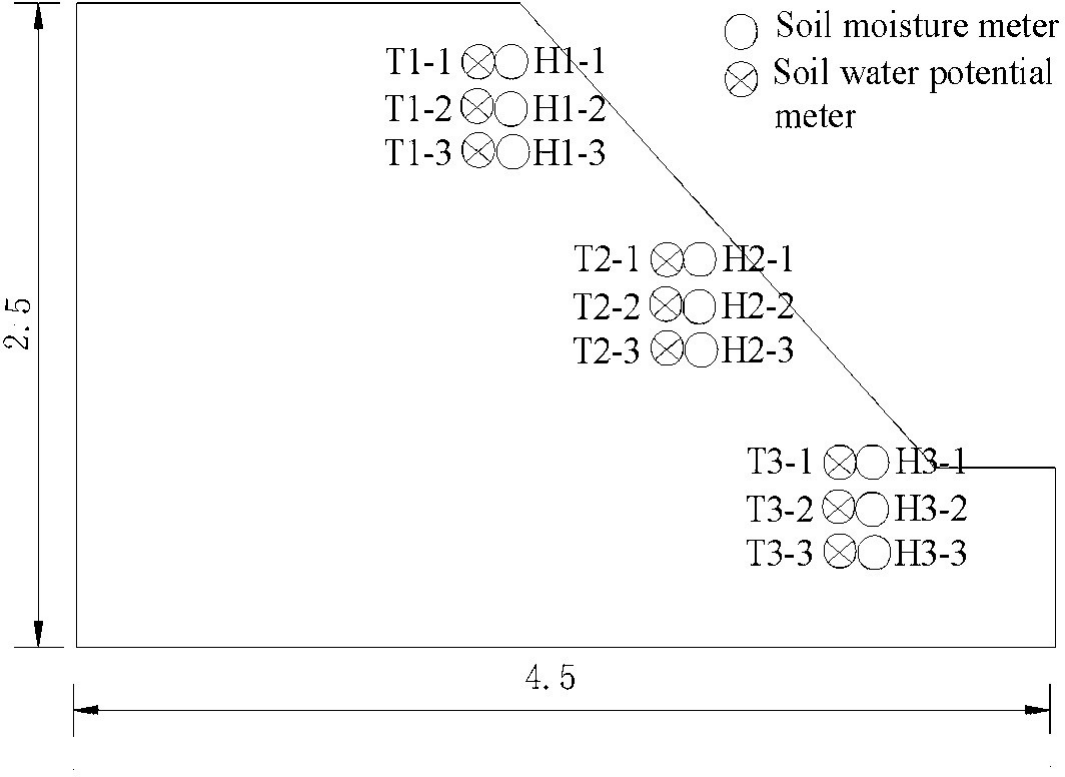
Schematic diagram of slope sensor embedding (unit: M).

**Fig 6:**
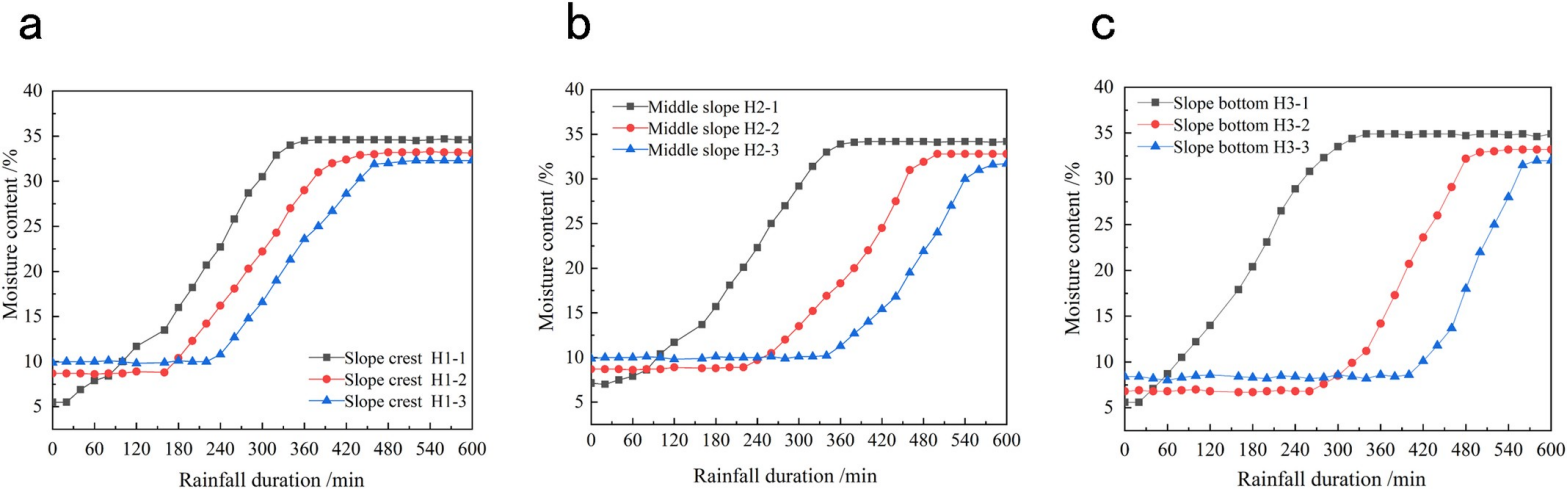
Relation curve between soil moisture content and rainfall time. **(a)** Time history curve of soil moisture content at the top of slope. **(b)** Time history curve of soil moisture content in the middle of slope. **(c)** Time history curve of soil moisture content at the bottom of slope.

*Monitoring the moisture content*. To monitor the slope moisture content during sustained rainfall, nine soil moisture sensors were buried at the top, middle, and toe of the model slope with burial depths of 0.2, 0.4, and 0.6 m from the slope surface, respectively. Fig 5 shows the embedding of the monitoring instruments. Fig 6 plots the relationship between soil moisture content and rainfall duration.

According to Fig 6A, the moisture content of the slope soil was almost unchanged within the first 20 min of sustained rainfall. Thereafter, the soil moisture content increased rapidly at H1-1, the measurement point nearest the slope surface. The increase rate slowed after 300 min of rainfall and was finally stabilized after 330 min of rainfall. At this time, the soil moisture content was 35.1%. In the middle (points H2-1, H2-2, and H2-3) and toe (points H3-1, H3-2, and H3-3) parts of the slope surface (Fig 6B and 6C, respectively), the soil moisture contents followed nearly the same trends as the top (point H1-1) of the slope, indicating that the rainfall intensity was smaller than the permeability coefficient of the soil mass and that the variations of soil moisture content were consistent across the slope surface. As the rainfall continued, the soil moisture contents at measurement points H1-2 and H1-3 increased rapidly after 160 and 220 min, respectively, and eventually stabilized at 33.6% and 32.1% after 400 and 450 min, respectively. The growth trends imply that the moisture content in the soil profile was gradually increased by rainfall infiltration and that the infiltration capability gradually decreased. Moreover, the soil mass expanded to shrink the potential cracks within the slope after water absorption, because coal soil contains montmorillonite. At this time, the rainfall infiltration slowed and lags were observed in the variations of the soil moisture contents at different depths. As the rainfall continued, the soil moisture content of the slope within a certain range was predicted to saturate. After rainwater discharge through the runoff of the slope, the soil moisture content tended to remain stable.

**Fig 7:**
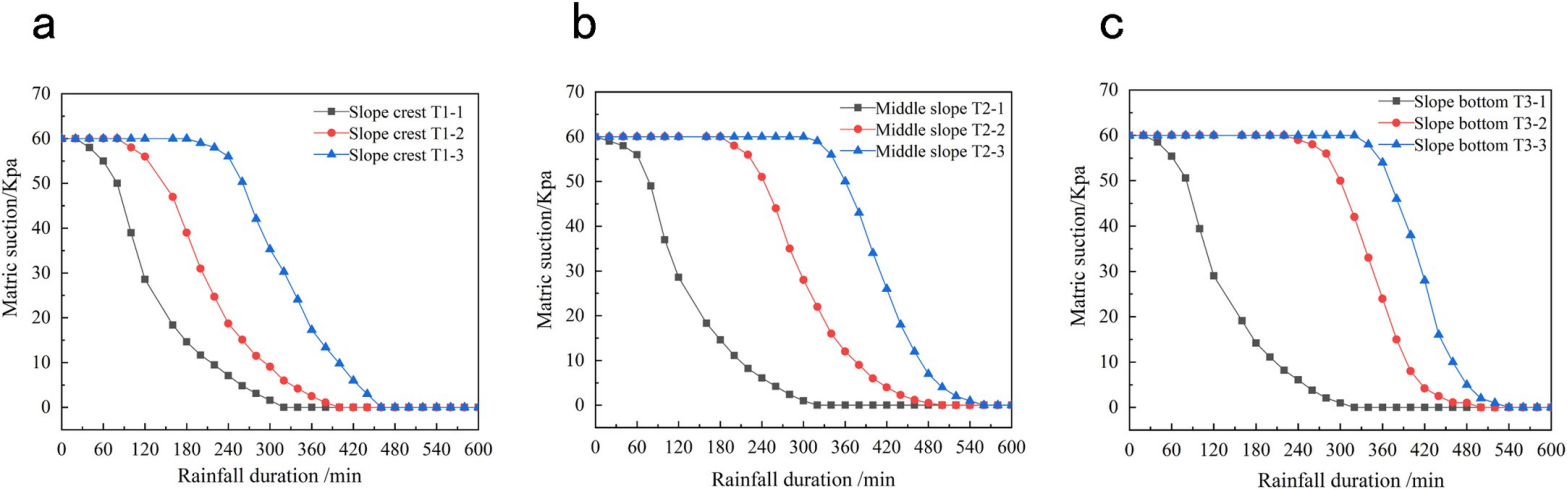
Curve of soil matric suction versus time. (a) Time history curve of soil matrix suction at the top of slope. (b) Time history curve of soil matrix suction in the middle of slope. (c) Time history curve of soil matrix suction at the bottom of slope.

*Monitoring the matrix suction*. To monitor the matrix suction of the soil during rainfall, nine soil–water potential testers were buried at the top, middle, and toe of the model slope with burial depths of 0.2, 0.4, and 0.6 m, respectively. Fig 5 shows the embedding of the monitoring instruments. Fig 7 plots the temporal changes in matrix suction obtained from the monitoring data.

As evidenced in Fig 7A, the soil matrix suction was nearly unchanged during the first 20 min of rainfall and then began declining at measuring point T1-1. As the rainfall continued, the infiltrated amount of rainwater increased while the matrix suction at measuring point T1-1 decreased rapidly. After 330 min of rainfall, the matrix suction at measuring point T1-1 dropped to 0. As the infiltration depth of the rainwater increased, the matrix suctions at measuring points T1-2 and T1-3 (at the top of the slope) began decreasing in succession. The variation times at the beginning and end were consistent with those of the soil moisture content at the same monitoring points. Meanwhile, the soil matrix suctions at the middle and toe of the slope (Fig 7B and 7C, respectively) varied similarly to that at the top of the slope. The results showed a direct negative correlation between the soil matrix suction and soil moisture content; that is, the soil matrix suction rapidly decreased with increasing moisture content. When the soil moisture content approached the saturation point, the matrix suction reduced to 0.

#### Numerical calculation model of the coal-bearing soil slope

**Fig 8:**
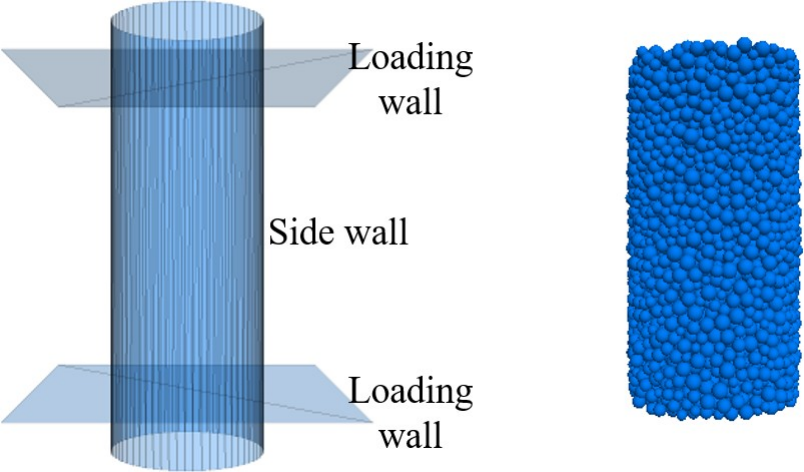
Numerical simulation model of triaxial test. (a) Boundary wall. (b) Particle wall.

*Model parameter calibration*. The meso-parameters were calibrated in a numerical simulation of a triaxial test constructed in PFC^3D^ software. The test model was a three-axis wall model with a height and a diameter of 4.0 and 2.0 m, respectively. To shorten the calculation time and improve the analysis efficiency, the actual soil particle size was enlarged and the processing was centralized. The particle size was randomly distributed between 50 and 90 mm, and the calculation model is shown in Fig 8.

**Fig 9:**
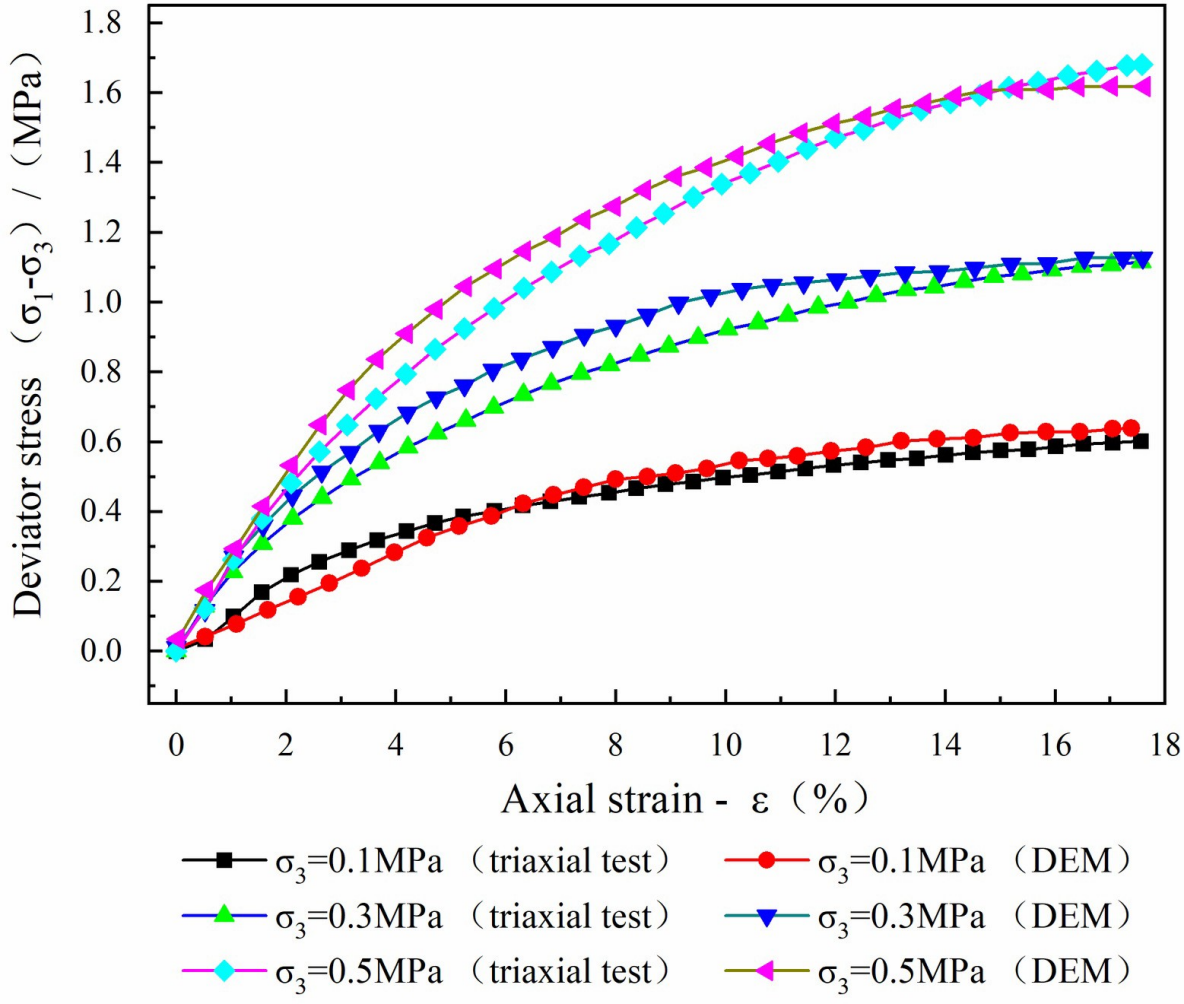
The meso parameters were calibrated by simulated triaxial test.

To capture the nature of coal-bearing soils, a contact bond model was selected for the numerical calibration and slope modeling. The contact bond model could not transfer the bending moments through the small contact points between the particles, even under normal and tangential stresses [35]. This situation mimics the properties of coal-bearing soils. The modeled particles were servo-loaded at confining pressures of 100, 300, and 500 KPa. After changing the microscopic parameters of the particles in repeated trial calculations, the deviating axial strain curves of the numerical simulations approached the laboratory test results. Fig 9 compares the numerically simulated curves with the laboratory test curves. The strong resemblance of the simulation curves to the laboratory results verifies that under the correct parameter settings, the model can microanalyze coal-bearing soils. The microcalibrated particle parameters (see Table 1) were used in subsequent numerical simulations of the slope.

**Table 1:** Microscopic parameters of coal bearing soil.

Friction	partical size(m)	$k_{n}^{cb}$(N/m)	$k_{s}^{cb}$(N/m)	*e*	dump	density(kg/m^3^)	E_mod_	k_ratio_
*μ*							(P_a_)	
0.5	0.05~0.09	4.5×10^4^	4.5×10^4^	0.35	0.7	2400	3×10^6^	2

**Fig 10:**
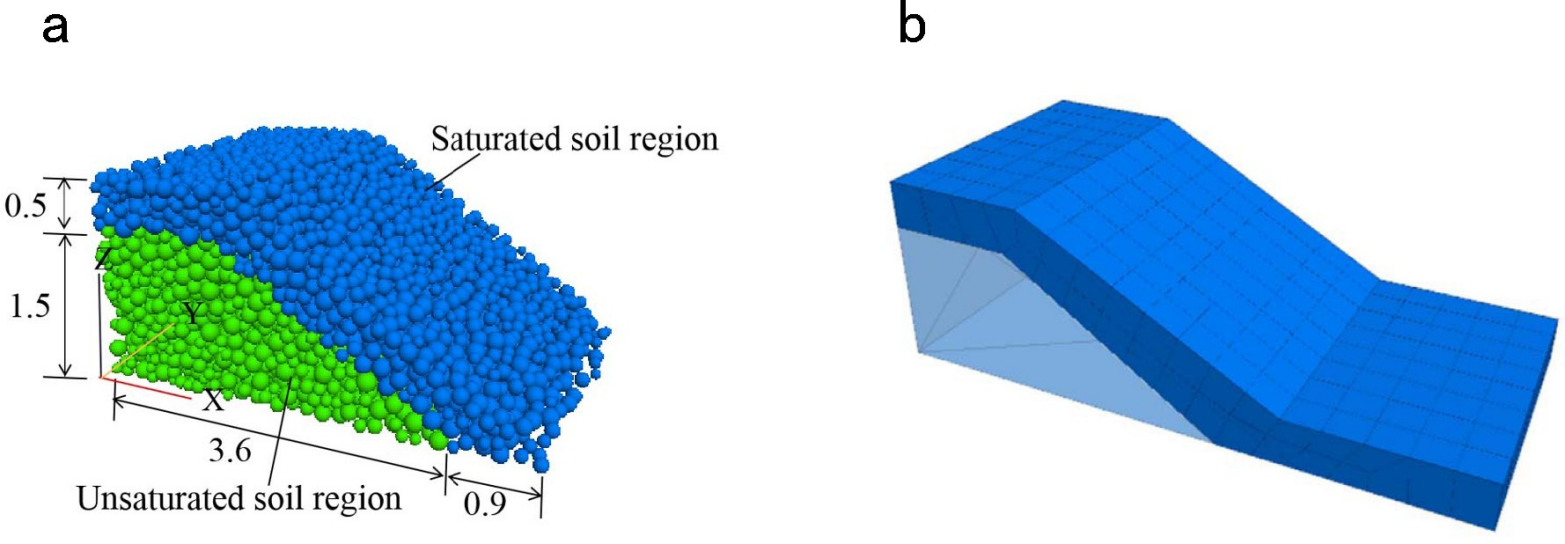
The fluid-solid coupling (CFD-DEM) calculation model of slope under rainfall infiltration. (a) The soil in the rainwater infiltration area is stratified. (b) Set up the seepage grid (unit: m).

*Establishment of the slope calculation model*. Fig 10 shows the 3D microscopic model established in the fish programming language for fluid–solid coupling (CFD–DEM) in sloped soil. In this model, we conducted an artificial rainfall test. As suggested in Fig 10A, the particles in the model were divided into two groups. The blue particles in the upper part (2646 particles) were in the rainwater-saturated zone of the slope and the light green particles in the lower part (4572 particles) occupied the unsaturated zone.

The simulation scenario was a model slope after an artificial rainfall of 8 h. The rainwater formed a transient saturated zone in the soil mass of the model slope, with an approximate penetration depth of 0.5 m (Fig 10A). To facilitate the modeling and calculation, the irregular boundary between the saturated and unsaturated soils was approximated by a straight line. Within the soil seepage area (Fig 10B), the fluid grid was extended along the front surface of the slope to match the real situation, as the particles moved down the slope with surface runoff after rainfall. In the unsaturated zone, the microscopic parameters of the particles were those of the triaxial test described above. In the saturated zone, where the soil strength was lower than in the unsaturated zone, the bond strength of the soil was reduced, whereas the other parameters were unchanged. Through the numerical simulation and parameter calibration of the triaxial test results of saturated coal measure soil, the normal bond strength and tangential bond strength of saturated coal measure soil are determined to be 1.5 × 10^4^N/m.

**Fig 11:**
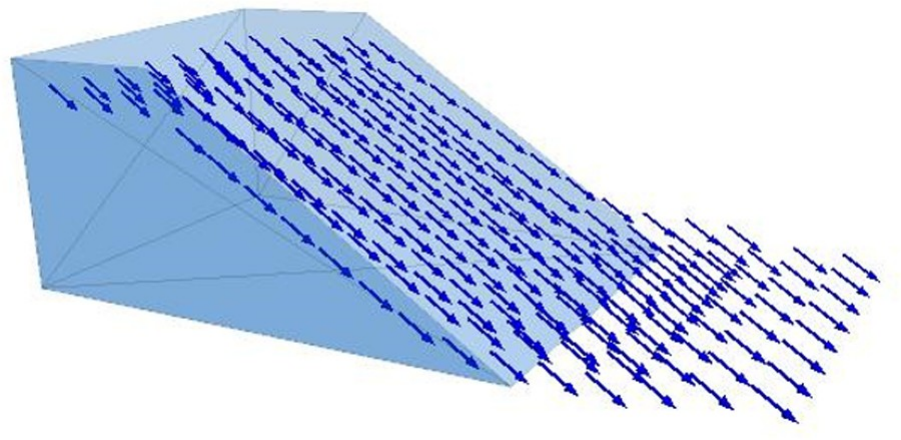
Direction of fluid movement in slope saturated area.

On the basis of the fluid characteristics of the model slope and the artificial rainfall intensity, the fluid parameters in the saturated zone of the slope were set to the values in Table 2. The ratio of flow rates along the *Z*-axis and *X*-axis was set to 1:1.5 to ensure consistency between the fluid direction and the actual flow (the effects of fluid flow along the *Y*-axis were ignored). As shown in Fig 11, the fluid flow direction was consistent with the slope–runoff direction in the outdoor rainfall test.

**Table 2:** Setting of fluid parameters in saturated soil.

density(kg/m^3^)	coefficient of viscosity	X-flow velocity(m/s)	Z-flow velocity(m/s)	Y-flow velocity(m/s)	mesh quantity
	(Pa·s)				
1000	0.001	0.33	-0.22	0	232

## Results

### Macroscopic analysis of particle movement in the coal-bearing soil slope

#### Analysis of calculated soil particle movement path on the slope

**Fig 12:**
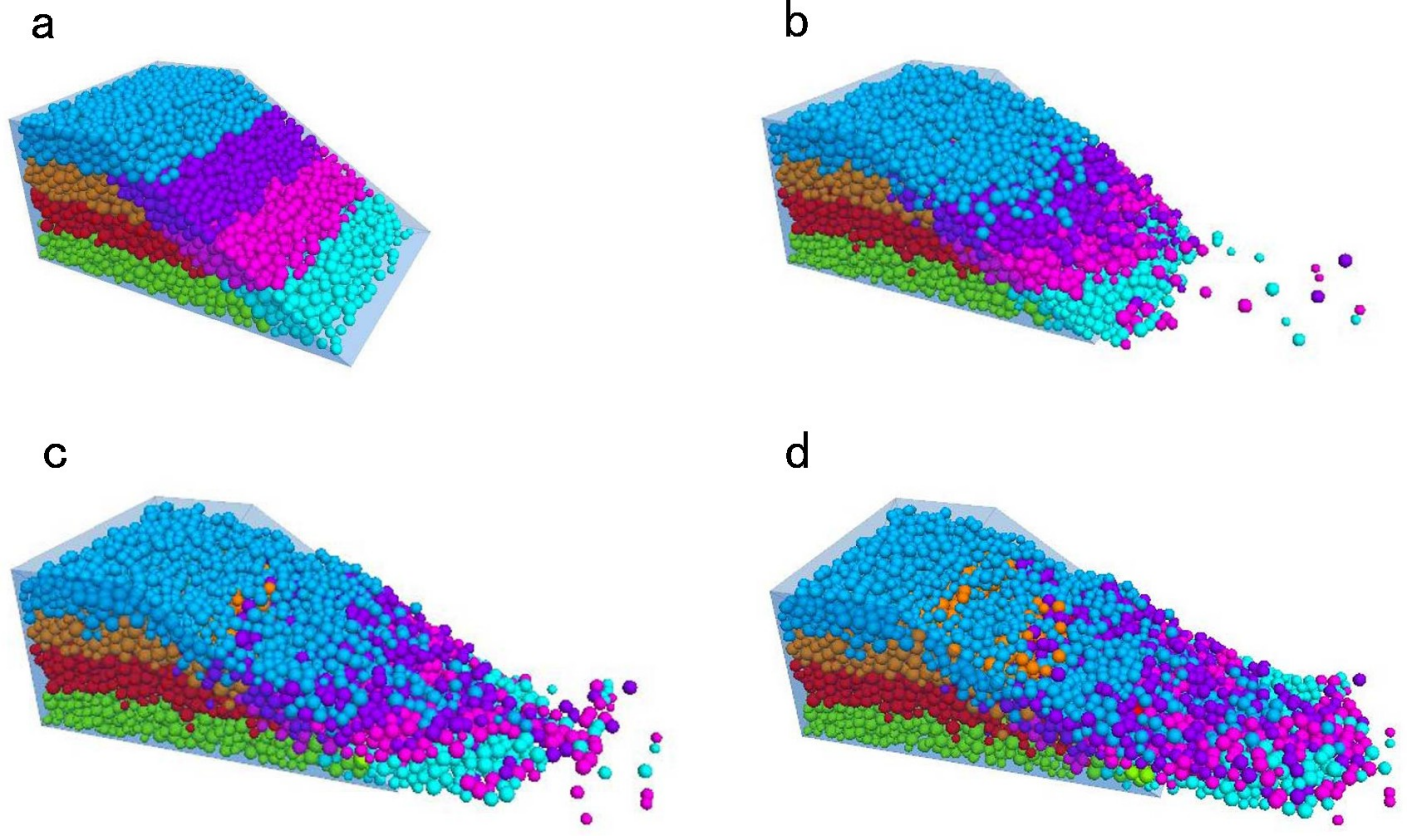
Particle movement of slope soil. (a) Initial state. (b) 20000 steps. (c) 60000 steps. (d) 100000 steps.

To observe the movement of soil particles at different locations on the wetted slope, the slope soil was examined in several areas. The saturated zone formed by rainwater infiltration was divided into four subzones, whereas the unsaturated zone was divided into three subzones (Fig 12). Under continuous action of the fluid, all particles were largely displaced from the upper slope and significantly accumulated at the slope toe (Fig 12). After 100,000 time steps, the model was essentially stable (see Fig 12D). At this time, the particles in the saturated zone were significantly shifted and most of the soil was washed from the slope. At the confluence of the saturated and unsaturated zones, some particles in the unsaturated zone of the slop mixed with those in the saturated zone. However, the soil particles in the unsaturated zone mainly remained stable, suggesting that rainwater infiltration caused significant damage only in the saturated zone of the slope.

**Fig 13:**
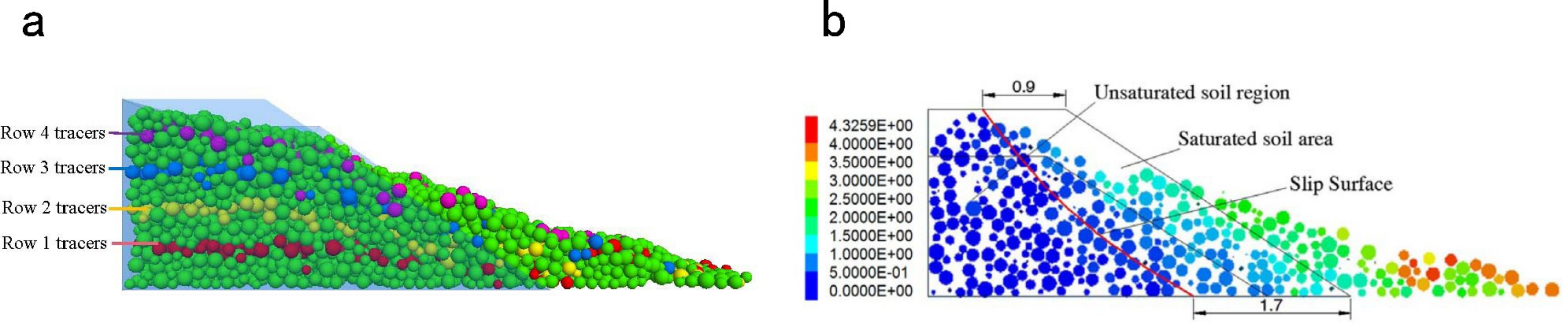
Schematic diagram of the overall particle movement of the slope. (a) Overall particle displacement diagram of slope. (b) Schematic diagram of slope sliding surface (unit: m).

Fig 13 shows the movement of soil particles on the side of the model after the simulation test. Particle displacement mainly occurred in the saturated zone and was local in the unsaturated zone (Fig 13A). The sliding zone in the particle displacement pattern (Fig 13B) was drawn with computer-aided design software, focusing on the middle section of the slope model. The upper and lower soil masses were displaced by approximately 0.9 and 1.7 m, respectively. The displacements accompanied the formation of an approximately linear sliding surface. The particles on the top of the slope were eroded to depths of 0.3–0.4 m, consistent with the test results of the outdoor slope model.

**Fig 14:**
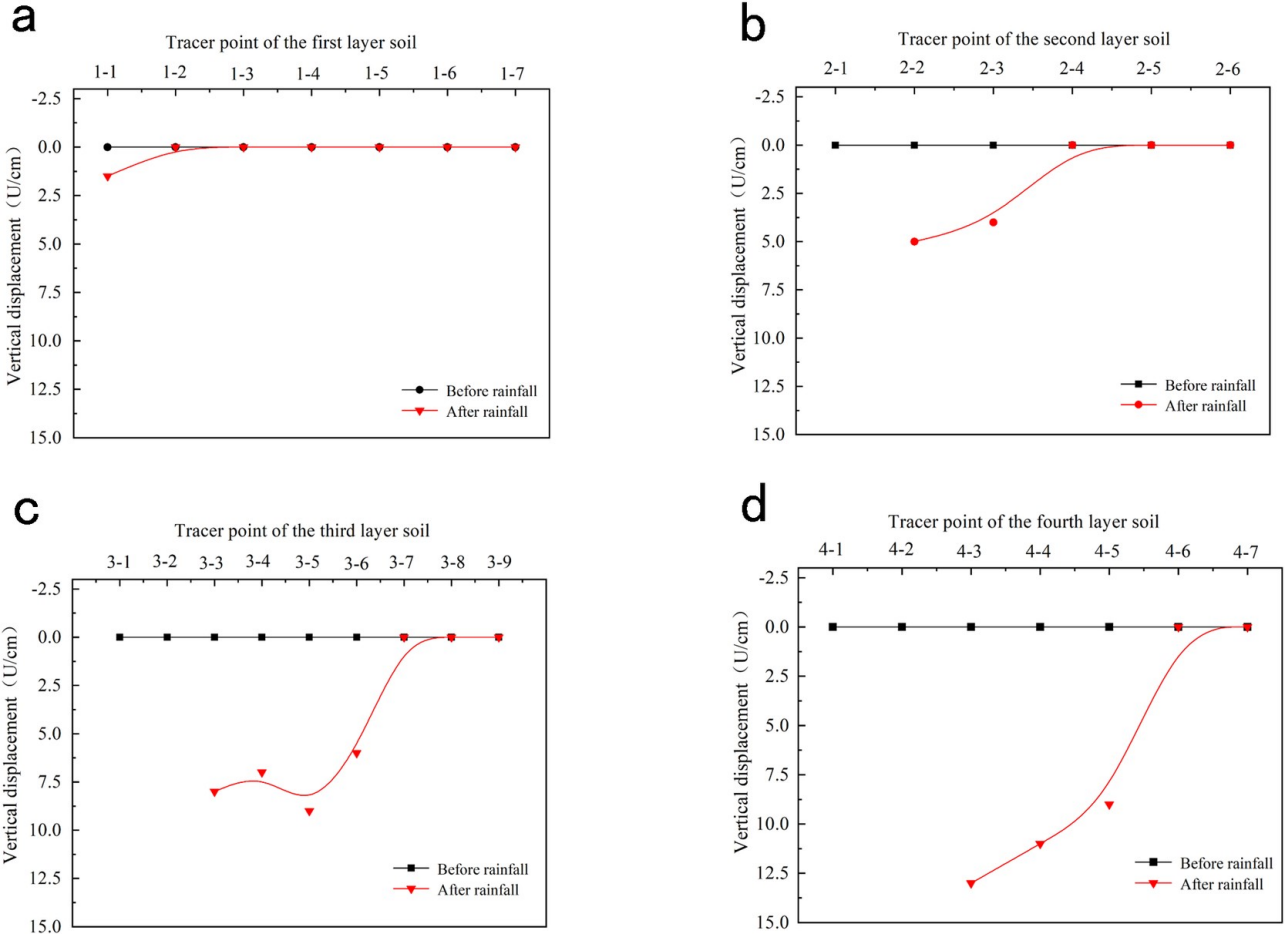
Comparison of the measured and simulated results of the track change of tracer points before and after rainfall. (a) The track change diagram of the tracer point of the first layer fill. (b) The track change diagram of the tracer point of the second layer fill. (c) The track change diagram of the tracer point of the third layer fill. (d) The track change diagram of the tracer point of the fourth layer fill.

To better observe the movements of the tracer points in the slope model during rainfall, Fig 14 plots the measured and simulated displacement changes of the tracer points before and after the rainfall (unless the tracer points had been washed away by rainwater). The number of tracer points in the figure is consistent with that in Fig 4. The numerical value in Fig 14 is the vertical displacement of the near-wall particles.

As shown in Fig 14, tracking points 4–1 and 4–2 on the fourth layer of the filling at the top of the slope were washed away by the rain. Tracking points 4–3, 4–4, and 4–5 were displaced by 13, 11, and 9 cm, respectively, in directions of 80° with respect to the ground. Tracking points 4–6 and 4–7 were almost unchanged during the rainfall. Tracking points 3–1 and 3–2 on the third layer of the filling were washed away, and tracking points 3–3, 3–4, 3–5, and 3–6 were displaced by 8, 7, 9, and 6 cm, respectively, in directions of 90° with respect to the ground. The rest of the tracking points on the third soil layer are unchanged during the rainfall process. Tracking point 2–1 on the second layer of the filling was washed away, and tracking points 2–2 and 2–3 were displaced by 5 and 4 cm, respectively, at directions of 90° with respect to the ground. The other tracking points in the second layer were unchanged during the rainfall process. Tracking point 1–1 on the first layer of filling was displaced by only ~1.5 cm, and the other tracking points in Layer 1 were unchanged. Meanwhile, tracking point 3–5 in the third layer of the filling was more displaced than the other tracking points in the same layer. This result indicates the presence of a crack in the soil, possibly within the sliding surface.

As evidenced in Fig 14, the numerically calculated displacement changes of the tracer points approached the measured values. Although the two sets of results differed in some aspects, their change trends were almost identical.

#### Analysis of calculated stress changes on the slope

**Fig 15:**
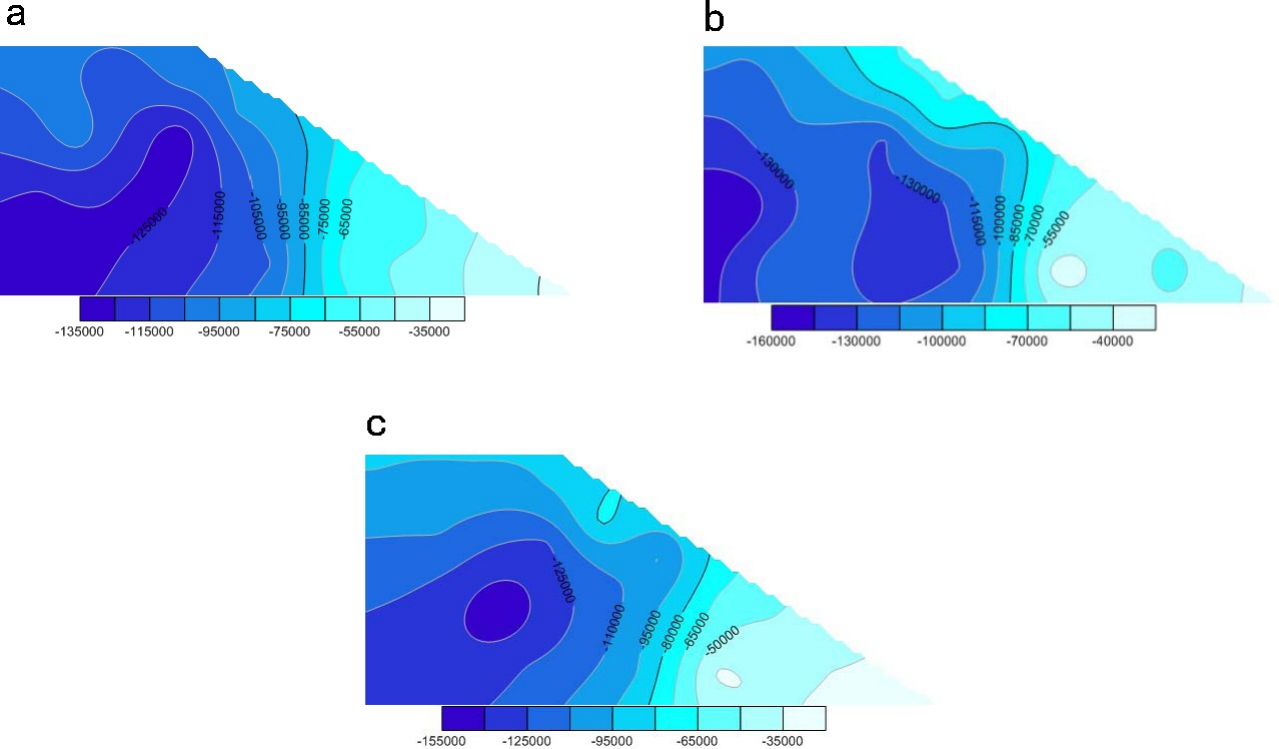
Cloud chart of stress change in each direction of slope at initial time. **(a)** Stress change of slope soil in X direction. **(b)** Stress change of slope soil in Z direction. **(c)** Stress change of slope soil in Y direction (unit: Pa).

**Fig 16:**
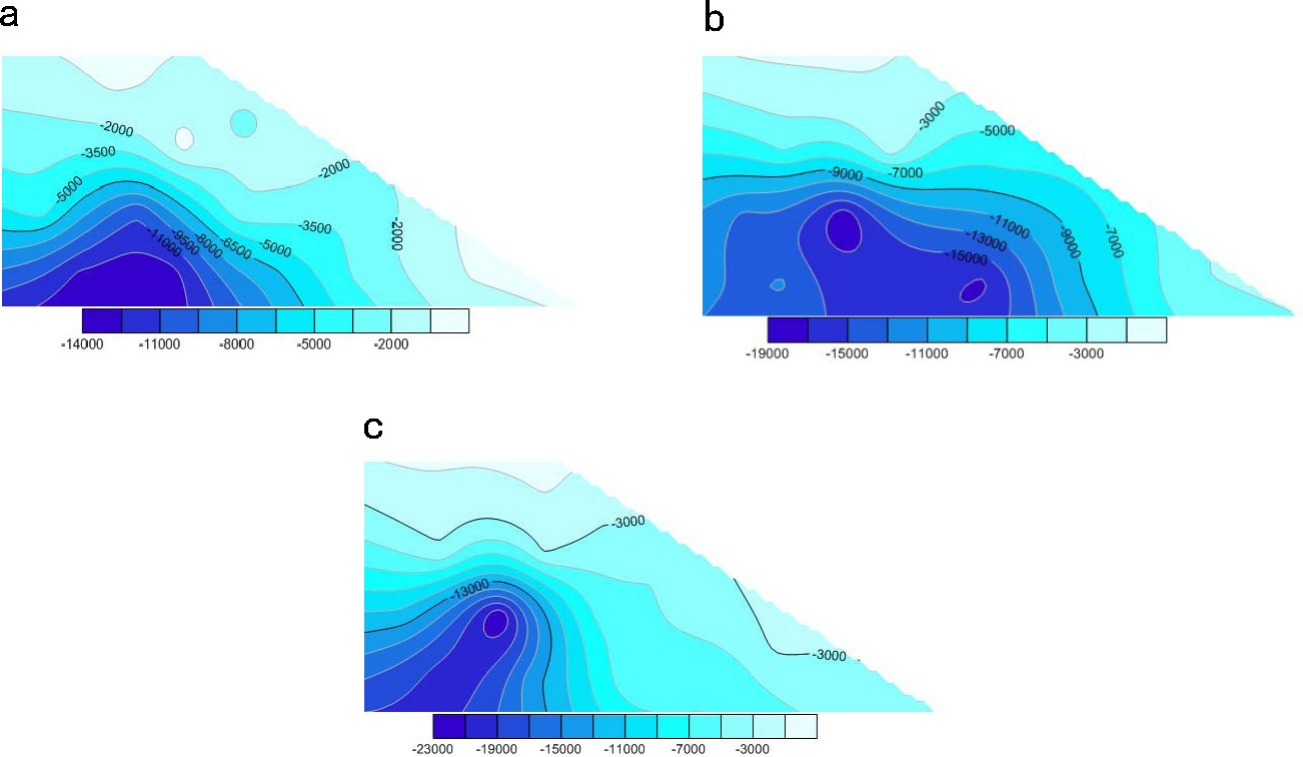
Cloud chart of stress change in each direction of slope soil when the model is calculated to 20000. **(a)** Stress change in X direction of slope at 20000 time steps. **(b)** Stress change in Z direction of slope at 20000 time steps. **(c)** Stress change in Y direction of slope at 20000 time steps (unit: Pa).

**Fig 17:**
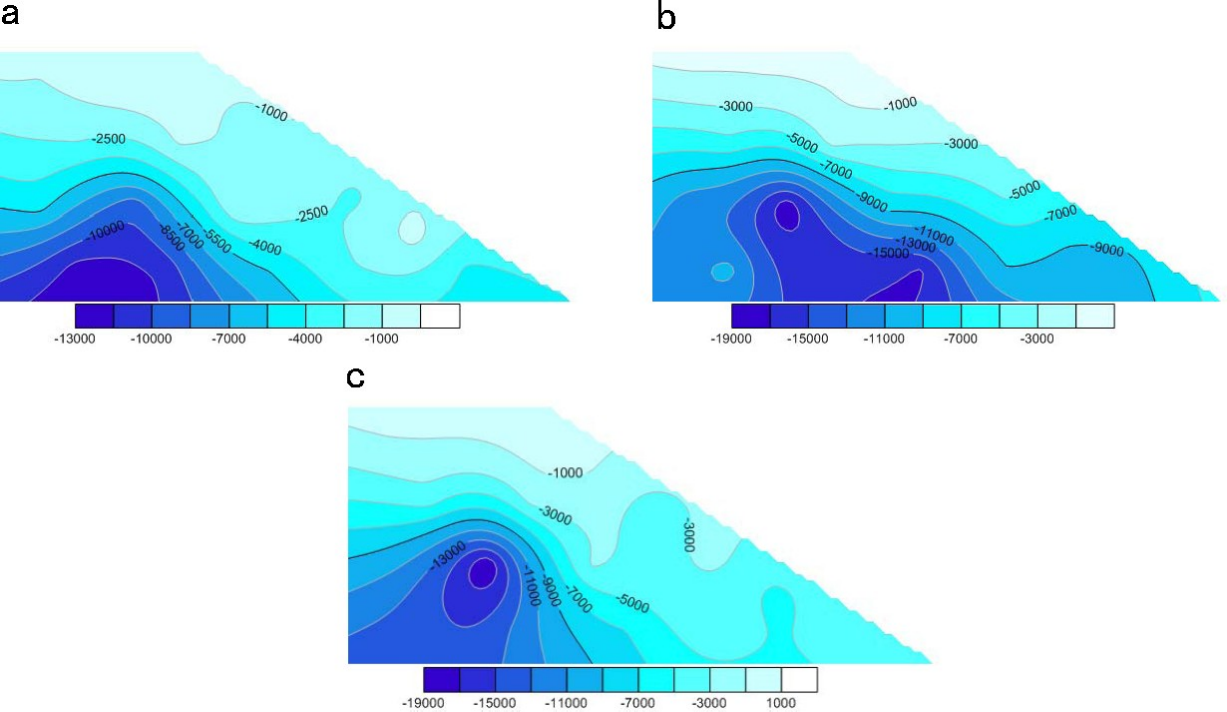
Cloud chart of stress change in each direction of slope soil when the model is calculated to 60000. **(a)** Stress change in X direction of slope at 60000 time steps. **(b)** Stress change in Z direction of slope at 60000 time steps. **(c)** Stress change in Y direction of slope at 60000 time steps (unit: Pa).

**Fig 18:**
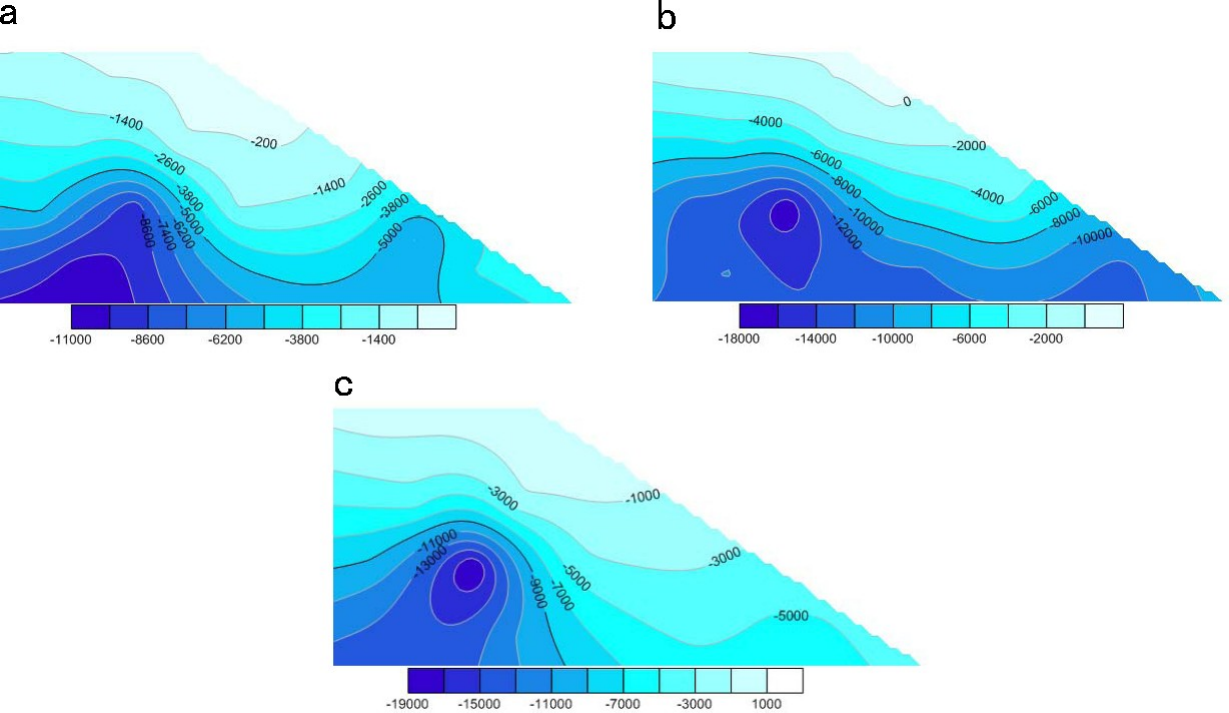
Cloud chart of stress change in each direction of slope soil when the model is calculated to 100000. (a) Stress change in X direction of slope at 100000 time steps. (b) Stress change in Z direction of slope at 100000 time steps. (c) Stress change in Y direction of slope at 100000 time steps (unit: Pa).

To identify the internal stress changes in the slope soil during rainfall infiltration and to analyze the soil failure mechanisms, 17 measuring circles were constructed in the simulated soil. After the fluid application, the monitored stress changes of the slope soil in the *X*, *Y*, and *Z* directions were simulated and processed in Surfer software to generate a cloud atlas. Figs 15–18 show the calculation results.

Comparing the stress evolution processes on the slope at each point, we concluded that (a) the walls at the top of the slope were not removed by rainfall. The interparticle contact force was high and the maximum soil stress in the *Z* direction was 0.16 MPa. The stresses were small on the top and bottom of the slope and large inside the slope (Fig 15). (b) After 20,000 time steps, the stress was lower on the upper slope than inside the slope and was mainly concentrated in the unsaturated zone (Fig 16). Under the action of the fluid, the total stress of the particles in the saturated zone decreased and the stability deteriorated. (c) After 60,000 time steps, the stress dramatically decreased on the top and upper part of the slope but increased at the slope toe. At this time, the interior of the slope was relatively stable, but the saturated zone was continuously damaged (Fig 17). (d) After 100,000 time steps, the stress had changed in all directions, mainly at the top and toe of the slope. The soil stress increased at the slope toe but significantly decreased on the top (see Fig 18). This result implies that rainwater conveyed the soil from the top to the toe of the slope, where many particles were accumulated.

### Micromechanism analysis of unstable failure in the coal-bearing soil slope

#### Analysis of particle force chain

**Fig 19:**
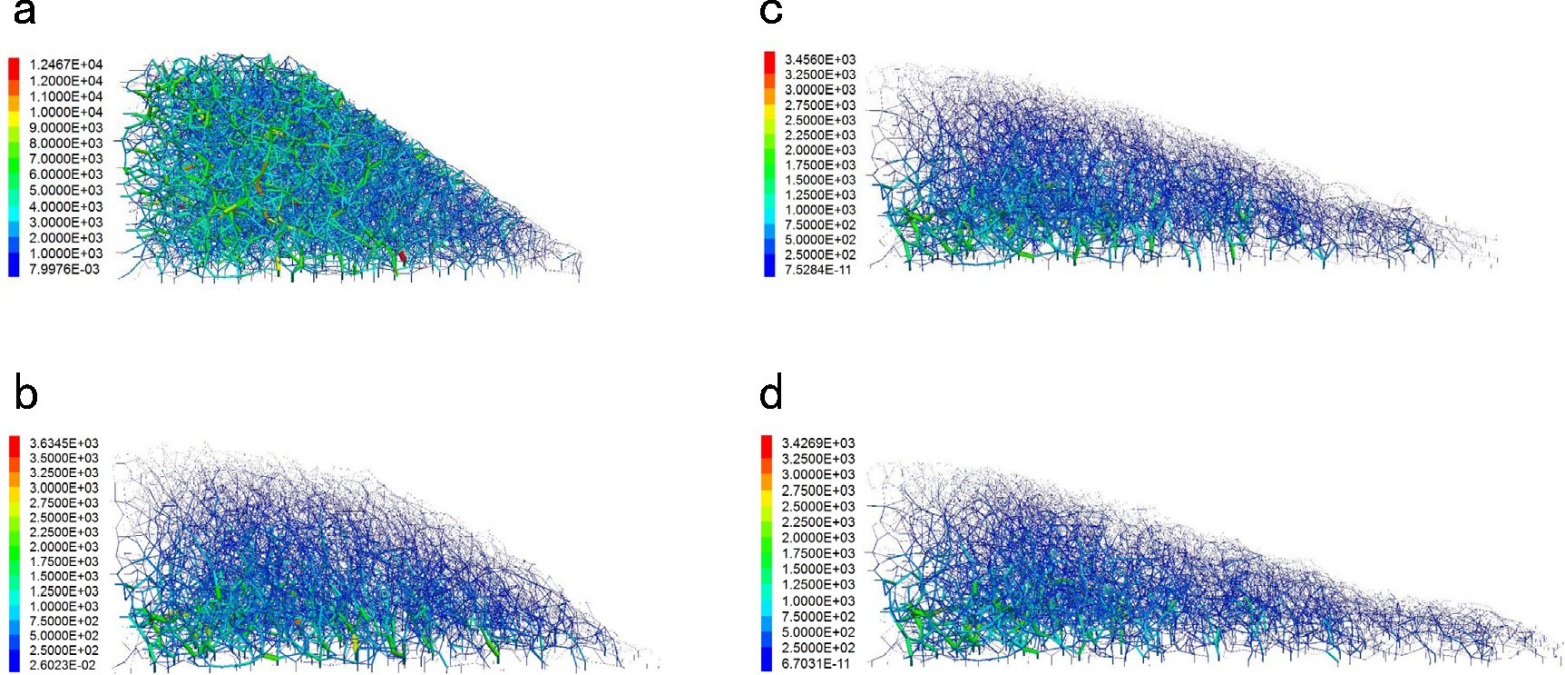
Evolution process of soil particle force chain in slope under rainfall. (a) The force chain of slope soil particles in initial state. (b) The force chain of soil particles in slope when the model calculated 20000 steps. (c) The force chain of soil particles in slope when the model calculated 60000 steps. (d) The force chain of soil particles in slope when the model calculated 100000 steps. (unit: Pa).

Fig 19 shows the force chain evolution of the slope model during rainfall. Under continuous rainfall, the force chain was sparse in the saturated zone of the slope, where the contact forces were small and the between-particle contacts were unstable. When fluid flowed in the upper slope, the force chain in the lower part of the unsaturated soil became dense and thick, suggesting a strong contact force between the particles in the slope and stable soil in that zone. Additionally, the force chain stretched significantly along the slope. As the gradient of the slope decreased, the force chain at the top of the slope reduced and became sparse, implying significant particle displacements in the saturated zone. After 100,000 time steps, the force chain of soil particles in the unsaturated zone remained almost constant (Fig 19D). By contrast, the particles on the slope surface of the saturated zone became sparse. This result indicates that the particles on the slope surface continued their downward movement and accumulated at the toe of the slope, which explains the thick and dense force chains at the slope toe and on the ground.

#### Analysis of changes in particle coordination number

**Fig 20:**
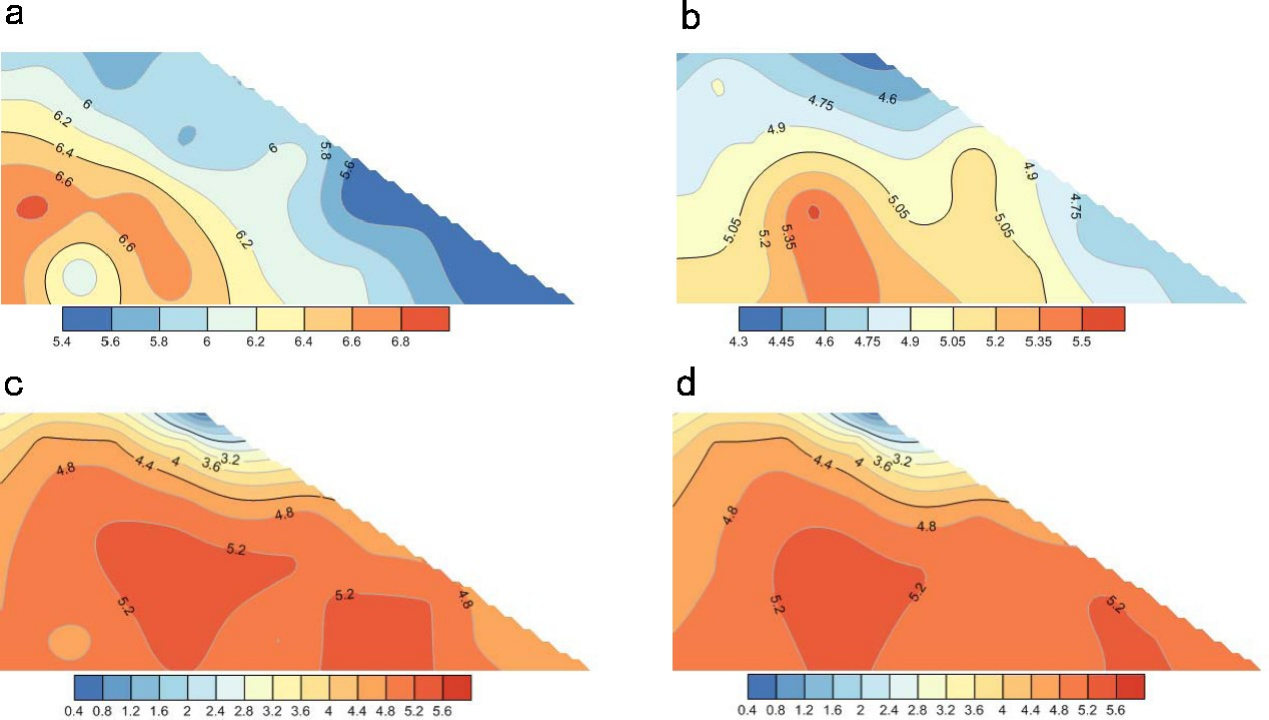
The change process of soil particle coordination number in slope under rainfall. (a) Distribution of particle coordination number of slope soil at initial time. (b) Distribution of soil particle coordination number in slope when the model calculated 20000 steps. (c) Distribution of soil particle coordination number in slope when the model calculated 60000 steps. (d) Distribution of soil particle coordination number in slope when the model calculated 100000 steps.

As shown in the cloud atlas of Fig 20, the coordination number of soil particles on the slope gradually decreased under continuous rainfall. In such circumstances, the coordination number of the soil particles was significantly reduced on the upper slope and the surface of the saturated zone and slightly reduced in the internal unsaturated zone. This result indicates weak contact and poor structural stability among the particles in the rain-affected areas, but fair contact and favorable stability among the particles in the unsaturated zone. After 60,000 time steps (Fig 20C and 20D), the coordination number decreased in the saturated zone (especially at the top of the slope) and increased in the interior and at the bottom of the slope. These observations imply that poor contact among the particles in the upper slope, the gradual downward movement of the particles, and their accumulation at the slope toe assured the compactness and good stability of the lower slope during the rainfall.

#### Analysis of particle porosity change

**Fig 21:**
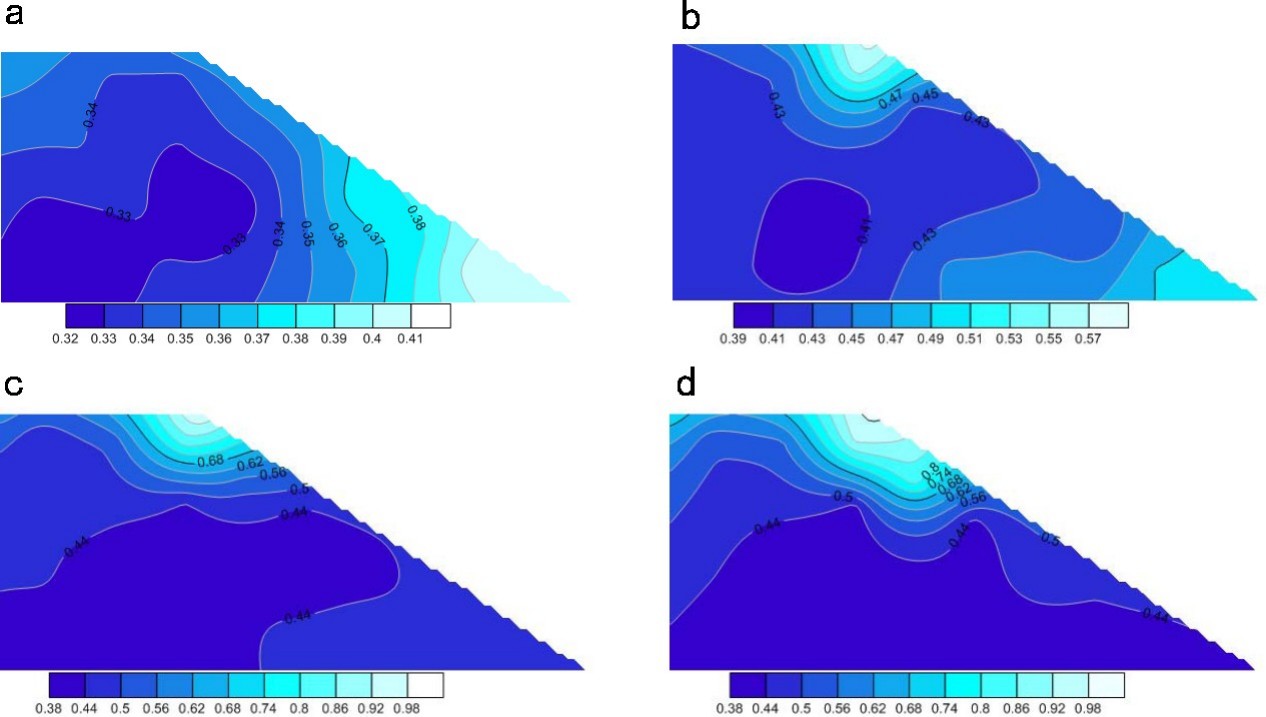
The change process of porosity of slope soil under rainfall. (a) Porosity distribution of slope soil in initial state. (b) Porosity distribution of slope soil when the model calculated 20000 steps. (c) Porosity distribution of slope soil when the model calculated 60000 steps. (d) Porosity distribution of slope soil when the model calculated 100000 steps.

Fig 21 shows the calculated porosity changes in the slope model during the rainfall. The soil porosity throughout the slope gradually increased under the continuous rainfall. The porosity was significantly increased in the saturated zone (mainly in the upper part of the slope) and less in the unsaturated zone. As shown in Fig 21C, the soil porosity at the toe of the slope slightly increased during the rainfall and remained constant after 60,000 time steps (the final porosity was 0.44). This means that under the influence of rainfall, the particles slid from the upper slope and accumulated at the toe, causing a slight change in soil porosity. By contrast, the porosity of the soil in the upper slope continued to increase, reaching 0.8 at the 100,000th time step (Fig 21D). This result indicates that under the influence of rainwater, the soil particles in the upper slope loosened and moved downward, which is consistent with the result in Fig 21C.

## Discussion

Coal-bearing soil slopes are easily weathered, softened, and disintegrated by water; accordingly, they can be treated as discontinuous media in DEM calculations and analyses. Comparing the DEM-simulated results with those of the outdoor rainfall test, we found that rainfall-induced erosion was crucially involved in the failure of coal-bearing soil slopes. Moreover, the slope–slip surface was an approximately linear segment. Finally, the erosion range of the model slope closely matched the slip surface predicted by the CFD–DEM coupling model. This study demonstrated that the CFD–DEM fluid–solid coupling method could rationally simulate the rainfall-induced failure of coal-bearing soil slopes. It also simulates Newton’s law of motion of discrete media in geotechnical engineering analyses [36].

The macroscopic mechanical properties of soil reflect the particle movement and positions, soil thickness, and density of the force chain, and intuitively represent the forces and movement changes of particles. During the rainfall, the force chain significantly stretched along the slope. As the slope gradient reduced, the force chains at the top and on the slope surface became sparse, implying instability and massive movement of the particles in the saturated zone. Under the combined action of fluid and particles in the upper slope, the force chain in the lower slope became dense and thick, indicating that large contact forces developed between the particles in the slope, jeopardizing the stability of the zone. The simulated force–chain changes revealed the failure evolution process of the slope under the influence of rainfall, providing a deep understanding of the failure mechanism of coal-bearing soil slopes.

The stability of slopes in discrete media can be analyzed in the CFD–DEM fluid–solid coupling model. Besides providing a theoretical basis for the protection design and construction of coal-bearing soil slopes in the study area, the results are expected to encourage analyses of macroscopic mechanical laws in discrete media from a micro–macro perspective in geotechnical engineering.

## Conclusions

In an outdoor rainfall test, erosion ditches were formed at different depths on the slope surface by rainwater washing, which is the main failure mode of slope soil. The ditches reached a maximum depth of 0.36 m in the mid-upper slope. The failure modes of coal-bearing soil slope were simulated in a CFD–DEM fluid–solid coupling model. The results were consistent with those of the outdoor rainfall test. The predicted slope–slip surface was approximately linear. The feasibility of applying the CFD–DEM fluid–solid coupling model to stability analyses of coal-bearing soil slopes was validated by the similarity between the simulated and actual model slope in the studied range of rainwater erosions.

During the rainfall, the changes in the microscopic parameters of the slope soil particles, such as the force chain, coordination number, and porosity, were directly related to the macroscopic mechanics of the slope soil. Thus, by analyzing the changes in the particle microparameters, we elucidated the failure evolution rules of coal-bearing soil slopes under the influence of rainwater. We also analyzed the failure mechanism of coal-bearing soil slopes (a type of discontinuous medium) from a micro–macro perspective.

The results verified that the stability of a discrete medium slope could be estimated by combining the DEM and the CFD method. The results not only provide a theoretical basis for the protection design and construction of coal-bearing soil slopes in this area but also provide new analyses of the macro mechanical laws of discrete media from the microperspective, which should advance the geotechnical engineering field.

## Supporting information

S1 File (PDF)
