# Supplementary material for: Macro and micro analysis on coal-bearing soil slopes instability based on CFD-DEM coupling method
Source: PLoS One. 2021 Sep 17;16(9):e0257362. doi: 10.1371/journal.pone.0257362 (PMC8448374; doi:10.1371/journal.pone.0257362)
Supplement: S1 File — (PDF) [file pone.0257362.s001.pdf]

Supporting information

S1 file

Calculation values of falling velocity of small balls with different sizes in liquid （m/s）

time step	Simulation results (particle size 1.5mm)	Simulation results (particle size 2.0mm)	Theoretical value (particle size 1.5mm)	Theoretical value (particle size 2.0mm)
0	0.00000	0.00000	-0.00686	-0.0121
50	-0.00235	-0.00255	-0.00686	-0.0121
100	-0.00391	-0.00458	-0.00686	-0.0121
150	-0.00494	-0.00618	-0.00686	-0.0121
200	-0.00561	-0.00744	-0.00686	-0.0121
250	-0.00605	-0.00843	-0.00686	-0.0121
300	-0.00634	-0.00921	-0.00686	-0.0121
350	-0.00653	-0.00981	-0.00686	-0.0121
400	-0.00666	-0.01030	-0.00686	-0.0121
450	-0.00674	-0.01070	-0.00686	-0.0121
500	-0.00679	-0.01100	-0.00686	-0.0121
550	-0.00683	-0.01120	-0.00686	-0.0121
600	-0.00685	-0.01140	-0.00686	-0.0121
650	-0.00686	-0.01150	-0.00686	-0.0121
700	-0.00687	-0.01160	-0.00686	-0.0121
750	-0.00688	-0.01170	-0.00686	-0.0121
800	-0.00688	-0.01180	-0.00686	-0.0121
850	-0.00689	-0.01180	-0.00686	-0.0121
900	-0.00689	-0.01190	-0.00686	-0.0121
950	-0.00689	-0.01190	-0.00686	-0.0121
1000	-0.00689	-0.01190	-0.00686	-0.0121
1050	-0.00689	-0.01190	-0.00686	-0.0121
1100	-0.00689	-0.01200	-0.00686	-0.0121
1150	-0.00689	-0.01200	-0.00686	-0.0121
1200	-0.00689	-0.01200	-0.00686	-0.0121
1250	-0.00689	-0.01200	-0.00686	-0.0121
1300	-0.00689	-0.01200	-0.00686	-0.0121

Monitoring values of moisture content change of slope top in outdoor test.

Rain duration (minute)	Moisture content at H1-1 of slope top (%)	Moisture content at H1-2 of slope top (%)	Moisture content at H1-3 of slope top (%)
0	5.5	8.7	9.9
20	5.5	8.7	10.0
40	6.9	8.7	10.0
60	7.9	8.6	10.0
80	8.4	8.7	10.1
100	10.0	8.7	10.0
120	11.7	8.9	9.8
160	13.5	8.8	9.9
180	16.0	10.4	10.1
200	18.2	12.3	10.0
220	20.7	14.2	10.0
240	22.7	16.2	10.8
260	25.8	18.1	12.7
280	28.7	20.3	14.8
300	30.5	22.2	16.6
320	32.9	24.3	19.0
340	34.0	27.0	21.3
360	34.5	29.0	23.6
380	34.6	31.0	25.0
400	34.6	32.0	26.7
420	34.6	32.4	28.6
440	34.6	32.9	30.3
460	34.6	33.0	31.9
480	34.6	33.2	32.0
500	34.6	33.2	32.2
520	34.5	33.2	32.3
540	34.6	33.3	32.3
560	34.7	33.2	32.3
580	34.6	33.2	32.3
600	34.6	33.1	32.3

Monitoring values of moisture content change in the middle of slope in outdoor test.

Rain duration (minute)	Moisture content at H2-1 of the middle of slope (%)	Moisture content at H2-2 of the middle of slope (%)	Moisture content at H2-3 of the middle of slope (%)
0	7.1	8.7	9.9
20	7.0	8.7	10.0
40	7.5	8.7	10.0
60	7.9	8.6	10.0
80	8.6	8.7	10.1
100	10.4	8.7	10.0
120	11.7	8.9	9.8
160	13.7	8.8	9.9
180	15.7	8.8	10.1
200	18.1	8.9	10.0
220	20.1	8.9	10.0
240	22.3	9.7	10.0
260	25.0	10.5	10.1
280	27.0	12.0	9.9
300	29.2	13.5	10.1
320	31.4	15.2	10.1
340	33.0	16.9	10.2
360	33.9	18.3	11.3
380	34.1	20.0	12.7
400	34.2	22.0	14.0
420	34.2	24.5	15.4
440	34.2	27.5	16.8
460	34.2	31.0	19.5
480	34.2	31.9	21.9
500	34.1	32.8	24.0
520	34.2	32.8	27.0
540	34.2	32.8	30.0
560	34.2	32.8	31.0
580	34.1	32.8	31.6
600	34.2	32.8	31.7

Monitoring values of moisture content change of the slope toe in outdoor test.

Rain duration (minute)	Moisture content at H3-1 of slope toe (%)	Moisture content at H3-2 of slope toe (%)	Moisture content at H3-3 of slope toe (%)
0	5.6	6.8	8.4
20	5.6	6.9	8.4
40	7.1	6.8	8.2
60	8.7	6.8	8.0
80	10.5	6.9	8.3
100	12.2	7.0	8.5
120	14.0	6.8	8.6
160	17.9	6.7	8.4
180	20.4	6.7	8.3
200	23.1	6.8	8.2
220	26.5	6.9	8.5
240	28.9	6.8	8.4
260	30.8	6.8	8.2
280	32.3	7.6	8.3
300	33.5	8.5	8.6
320	34.4	9.9	8.4
340	34.9	11.2	8.2
360	34.9	14.2	8.6
380	34.9	17.3	8.4
400	34.8	20.7	8.6
420	34.9	23.6	10.1
440	34.9	26.0	11.8
460	34.9	29.1	13.7
480	34.7	32.2	18.0
500	34.9	32.9	22.0
520	34.9	33.0	25.0
540	34.8	33.2	28.0
560	34.9	33.2	31.5
580	34.6	33.2	32.0
600	34.9	33.2	32.0

Monitoring values of matrix suction change on the top of slope in outdoor test.

Rain duration (minute)	Matric suction at H1-1 of slope top (Kpa)	Matric suction at H1-2 of slope top (Kpa)	Matric suction at H1-3 of slope top (Kpa)
0	60.0	60.0	60.0
20	60.0	60.0	60.0
40	58.0	60.0	60.0
60	55.0	60.0	60.0
80	50.0	60.0	60.0
100	39.0	58.0	60.0
120	28.6	56.0	60.0
160	18.4	47.0	60.0
180	14.6	39.0	60.0
200	11.7	31.0	59.0
220	9.5	24.7	58.0
240	7.1	18.7	56.0
260	4.8	15.1	50.3
280	3.1	11.5	42.1
300	1.6	9.1	35.3
320	0.0	6.0	30.3
340	0.0	4.2	24.1
360	0.0	2.5	17.3
380	0.0	1.1	13.4
400	0.0	0.0	9.8
420	0.0	0.0	6.0
440	0.0	0.0	3.0
460	0.0	0.0	0.0
480	0.0	0.0	0.0
500	0.0	0.0	0.0
520	0.0	0.0	0.0
540	0.0	0.0	0.0
560	0.0	0.0	0.0
580	0.0	0.0	0.0
600	0.0	0.0	0.0

Monitoring values of matrix suction change in the middle of slope in outdoor test.

Rain duration (minute)	Matric suction at H2-1 of the middle of slope (Kpa)	Matric suction at H2-2 of the middle of slope (Kpa)	Matric suction at H2-3 of the middle of slope (Kpa)
0	60.0	60.0	60.0
20	59.0	60.0	60.0
40	58.0	60.0	60.0
60	56.0	60.0	60.0
80	49.0	60.0	60.0
100	37.0	60.0	60.0
120	28.6	60.0	60.0
160	18.3	60.0	60.0
180	14.6	60.0	60.0
200	11.1	58.0	60.0
220	8.2	56.0	60.0
240	6.1	51.0	60.0
260	4.2	44.0	60.0
280	2.4	35.0	60.0
300	1.0	28.0	60.0
320	0.0	22.0	59.0
340	0.0	16.0	56.0
360	0.0	12.0	50.0
380	0.0	9.0	43.0
400	0.0	6.0	34.0
420	0.0	4.0	26.0
440	0.0	2.3	18.0
460	0.0	1.2	12.0
480	0.0	0.5	7.0
500	0.0	0.0	4.0
520	0.0	0.0	2.0
540	0.0	0.0	1.0
560	0.0	0.0	0.0
580	0.0	0.0	0.0
600	0.0	0.0	0.0

Monitoring values of matrix suction change of slope toe in outdoor test.

Rain duration (minute)	Matric suction at H3-1 of slope toe (Kpa)	Matric suction at H3-2 of slope toe (Kpa)	Matric suction at H3-3 of slope toe (Kpa)
0	60.0	60.0	60.0
20	60.0	60.0	60.0
40	58.5	60.0	60.0
60	55.4	60.0	60.0
80	50.6	60.0	60.0
100	39.4	60.0	60.0
120	29.0	60.0	60.0
160	19.1	60.0	60.0
180	14.2	60.0	60.0
200	11.1	60.0	60.0
220	8.2	60.0	60.0
240	6.1	59.0	60.0
260	3.8	58.0	60.0
280	2.1	56.0	60.0
300	1.0	50.0	60.0
320	0.0	42.0	60.0
340	0.0	33.0	58.0
360	0.0	24.0	54.0
380	0.0	15.0	46.0
400	0.0	8.0	38.0
420	0.0	4.2	28.0
440	0.0	2.5	16.0
460	0.0	1.1	10.0
480	0.0	1.0	5.0
500	0.0	0.0	2.0
520	0.0	0.0	1.0
540	0.0	0.0	0.0
560	0.0	0.0	0.0
580	0.0	0.0	0.0
600	0.0	0.0	0.0

Numerical simulation of triaxial test for mesoscopic parameter calibration.(unit:Mpa)

Axial strain / ε（%）	σ3=0.1MPa（triaxial test）	σ3=0.1MPa（DEM）	σ3=0.3MPa（triaxial test）	σ3=0.3MPa（DEM）	σ3=0.5MPa（triaxial test）	σ3=0.5MPa（DEM）
0.00	0.0000	0.00660	0.0000	0.01158	0.0000	0.03502
0.53	0.0333	0.04176	0.1287	0.11662	0.1209	0.17489
1.04	0.0991	0.07812	0.2273	0.27116	0.2632	0.29379
1.56	0.1692	0.11825	0.3081	0.36061	0.3798	0.41543
2.09	0.2188	0.15513	0.3804	0.44565	0.4828	0.53267
2.60	0.2567	0.19386	0.4404	0.51474	0.5721	0.64906
3.15	0.2894	0.23735	0.4938	0.57016	0.6492	0.74909
3.66	0.3186	0.28309	0.5404	0.63112	0.7241	0.83684
4.19	0.3441	0.32544	0.5844	0.68383	0.7953	0.90974
4.71	0.3678	0.35854	0.6253	0.72702	0.8652	0.98014
5.22	0.3867	0.38748	0.6628	0.76209	0.9243	1.04559
5.79	0.4019	0.42359	0.6989	0.80618	0.9833	1.09589
6.31	0.4173	0.44806	0.7355	0.83759	1.0409	1.14642
6.81	0.4294	0.46984	0.7665	0.87162	1.0866	1.18729
7.34	0.4421	0.49294	0.7963	0.90654	1.1327	1.23712
7.88	0.4538	0.50090	0.8212	0.93259	1.1674	1.27421
8.38	0.4667	0.50999	0.8488	0.96351	1.2139	1.32134
8.91	0.4783	0.52356	0.8745	0.99831	1.2546	1.36069
9.41	0.4864	0.54595	0.8981	1.01897	1.3004	1.38672
9.95	0.4977	0.55204	0.9228	1.03771	1.3388	1.41812
10.45	0.5049	0.55972	0.9414	1.04897	1.3706	1.45426
10.95	0.5145	0.57368	0.9626	1.05608	1.4037	1.48549
11.45	0.5237	0.58387	0.9858	1.06498	1.4391	1.51254
11.96	0.5331	0.60244	1.0006	1.07537	1.4717	1.53114
12.46	0.5407	0.60822	1.0182	1.08538	1.4944	1.55527
12.96	0.5481	0.61287	1.0357	1.08802	1.5248	1.57011
13.48	0.5531	0.62575	1.0441	1.09882	1.5506	1.59067
14.00	0.5622	0.62882	1.0598	1.11033	1.5708	1.60731
14.50	0.5694	0.62891	1.0733	1.11148	1.5933	1.60994
15.01	0.5751	0.63753	1.0804	1.12699	1.6168	1.60918
15.51	0.5783	0.63921	1.0923	1.12797	1.6308	1.61693
16.01	0.5867	0.63924	1.1033	1.12794	1.6499	1.61968
16.51	0.5947	0.63936	1.1073	1.12793	1.6619	1.61851
17.05	0.5979	0.63962	1.1153	1.12796	1.6779	1.61936
17.56	0.6023	0.63917	1.1157	1.12795	1.6818	1.61953
